# Towards allosteric receptors – synthesis of β-cyclodextrin-functionalised 2,2’-bipyridines and their metal complexes

**DOI:** 10.3762/bjoc.10.77

**Published:** 2014-04-09

**Authors:** Christopher Kremer, Gregor Schnakenburg, Arne Lützen

**Affiliations:** 1 University of Bonn, Kekulé-Institute of Organic Chemistry and Biochemistry, Gerhard-Domagk-Str. 1, D-53121 Bonn, Germany; 2 University of Bonn, Institute of Inorganic Chemistry, Gerhard-Domagk-Str. 1, D-53121 Bonn, Germany

**Keywords:** allosteric receptors, 2,2’-bipyridines, β-cyclodextrin, supramolecular chemistry, metal complexes

## Abstract

Herein, we present three new 2,2’-bipyridines that carry two β-cyclodextrin moieties in different substitution patterns. When coordinated by zinc(II) or copper(I) ions (or their complexes), these compounds undergo conformational changes and switch between “open” and “closed” forms and thereby bringing together or separating the cyclodextrin moieties from each other.

## Introduction

In biological systems, recognition events play a major role to guarantee the selectivity of essential processes. Multivalent [1–2] and cooperative binding [3–5] are two of the major concepts to ensure and control the efficiency in those events and the associated complex biochemical cascades following. In order to regulate several of their metabolical functions, a process named “allosteric regulation” is used by enzymes and proteins [6], which stands for cooperative effects in the binding of more than one substrate to different sites of a receptor. After binding of the first substrate (the effector), the receptor changes its conformation, and by this enhancing (positive allosteric cooperativity) or hampering (negative allosteric cooperativity) the binding of another substrate at a second binding site.

This powerful regulatory concept has become quite interesting in supramolecular chemistry, and the development of artificial receptor systems which can be controlled by allosteric effects comes out of it [7–14].

2,2’-Bipyridines have been demonstrated to be excellent artificial allosteric centres due to their well-known ability to switch between *syn*- and *anti*-conformations as an answer to an external stimulus [15]. This switching process can be controlled either by adding or removing suitable transition metal ions or their complexes [16] or by adjusting the pH value [17–19]. The latter, however, is not as secure as using metal ions because of three possible protonation states (not protonated: *anti*, protonated once: *syn*, protonated twice: *anti*) instead of two states using metal ions (not coordinated: *anti*, coordinated: *syn*) and was not used in our work for this reason.

**Scheme 1:**
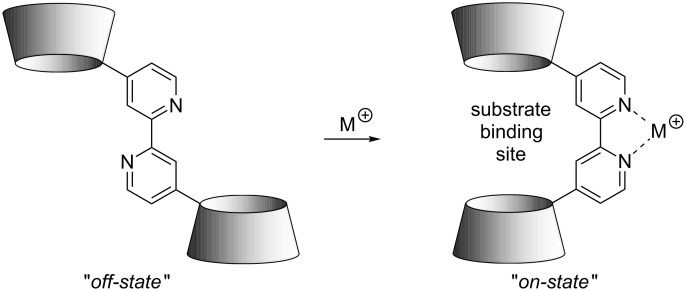
*Off*- (open) and *on*- (closed) states of a ditopic positive allosteric receptor based on a 4,4’-functionalised 2,2’-bipyridine.

Some time ago we developed a series of 2,2’-bipyridine-based allosteric analogues [20–22] of the well-known resorcinarene-based hemicarcerands [23–24]. However, these possess only rather shallow binding sites that bind non-polar substrates via dispersive interactions. Hence, we wanted to take this approach one step further by using β-cyclodextrins as another class of macrocyclic compounds that are very well-known for their excellent recognition properties towards non-polar substrates [25]. Depending on the substitution pattern for the central 2,2’-bipyridines, three conceptually different types of allosteric receptors can be obtained: 4,4’- or 6,6’-substitution, both leading to positive allosteric receptors, meaning that they adopt an open *anti*-conformation in the free state where the two cyclodextrins cannot interact with a substrate in a cooperative fashion (*off*-state) but change their conformation to a closed *syn*-conformation (*on*-state) upon binding of a suitable transition metal ion or complex as an effector. This is shown schematically for a 4,4’-substituted bipyridine-based receptor in Scheme 1.

**Scheme 2:**
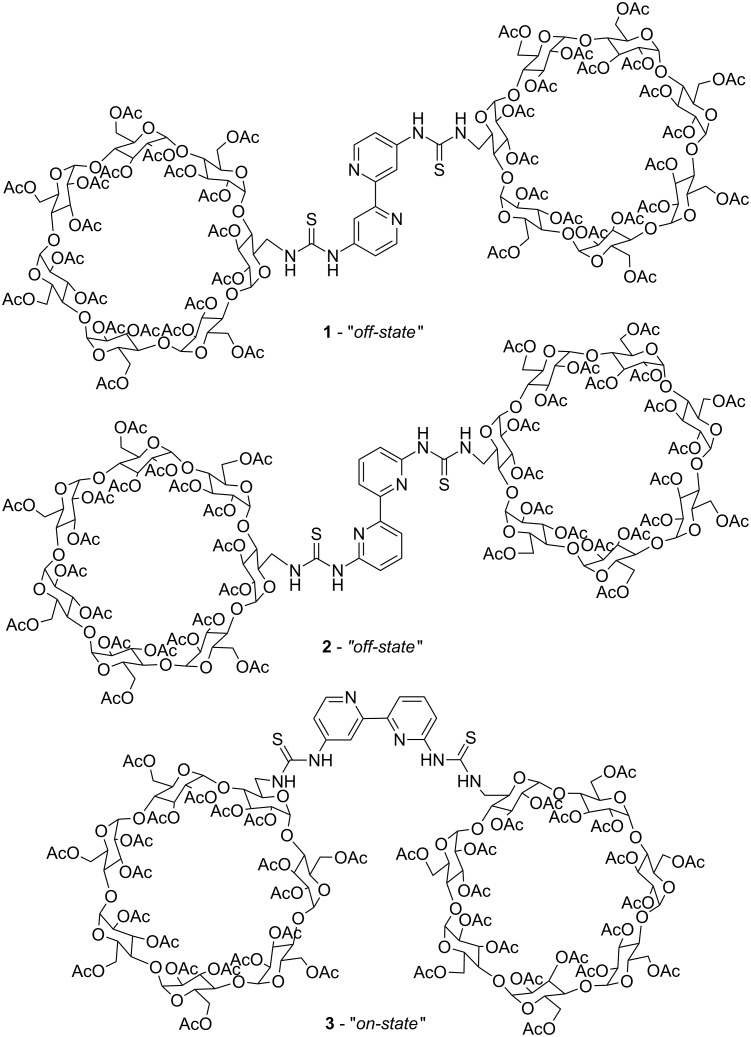
Bis(β-cyclodextrin)-functionalised 2,2’-bipyridines **1**–**3**.

However, these two scenarios differ in the fact that the effector is bound close to the substrate in case of a 6,6’-substitution whereas it is bound in a remote place in case of a 4,4’-substitution. In contrast, a 4,6’-substitution leads to a negative allosteric system which adopts the closed *on*-state in the absence of the effector and changes its conformation to the open *off*-state upon binding of it. In this article we present the synthesis of three new cyclodextrin-functionalised 2,2’-bipyridines **1**–**3** differing in the bipyridine’s substitution pattern (Scheme 2) and their metal complexes.

## Results and Discussion

### Synthesis

The synthesis of **1**–**3** requires the three different 2,2’-bipyridines that carry isothiocyanate functions and a monoaminated β-cyclodextrin that could be coupled with each other via two-fold nucleophilic addition.

**Scheme 3:**
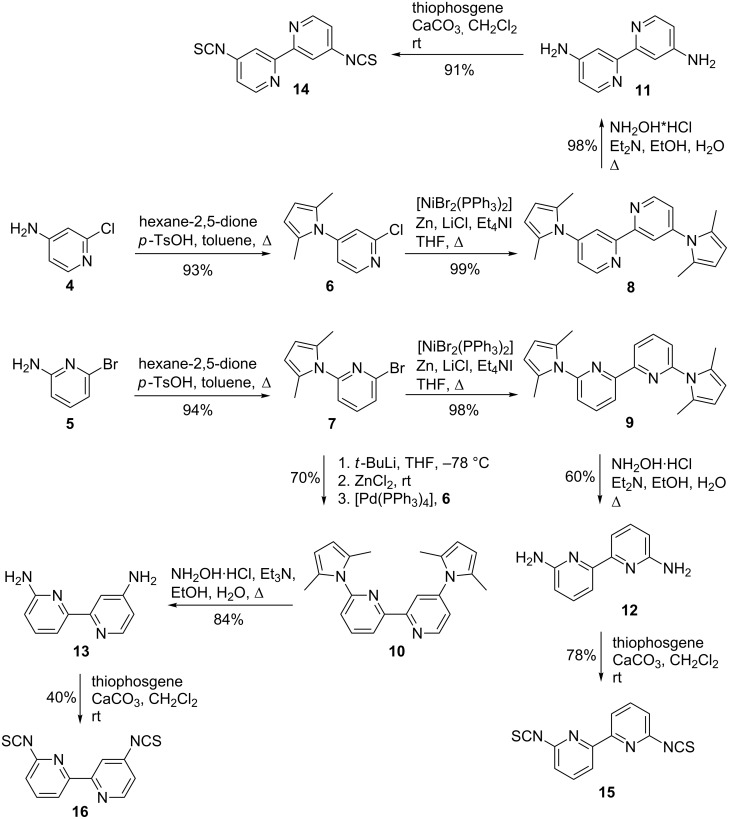
Synthesis of diisothiocyanato-2,2’-bipyridines **14**–**16**.

Symmetrically 4,4’- and 6,6’-disubstituted 2,2’-bipyridines were prepared starting from commercially available 4-amino-2-chloropyridine (**4**) and 2-amino-6-bromopyridine (**5**), respectively. After protection of the amino functions as 2,5-dimethylpyrroles, pyridines **6** and **7** were transformed into the corresponding 2,2’-bipyridines **8** and **9** via a nickel-catalysed homo-coupling reaction in excellent yields [26]. The non-symmetric 4,6’-disubstituted bipyridine **10** was obtained in good yield from a Negishi cross-coupling of **6** and **7** [27]. Cleavage of the pyrrole protecting groups of **8**–**10** with hydroxylamine provided the corresponding diamines **11**–**13** [26] in satisfying to excellent yields which were finally reacted with thiophosgene to give rise to the desired diisothiocyanates **14**–**16** (Scheme 3) [28].

**Scheme 4:**
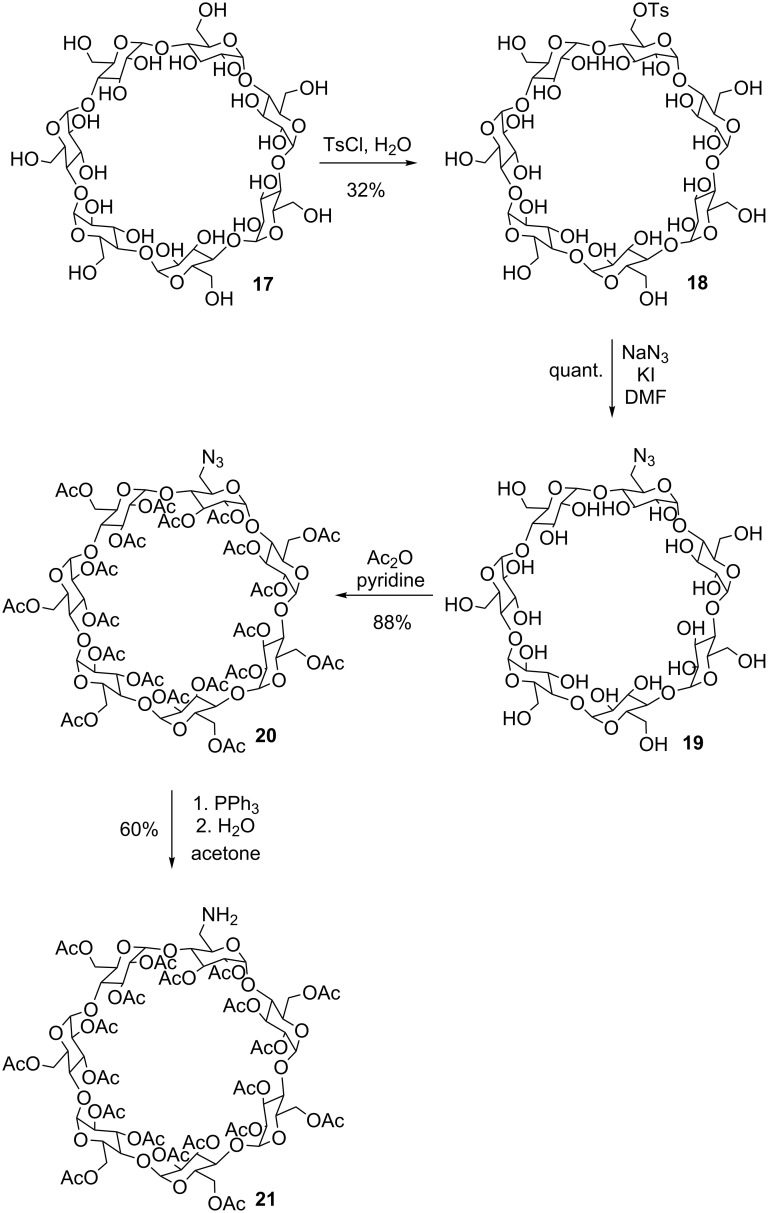
Synthesis of peracetylated cyclodextrin **21**.

β-Cyclodextrin (**17**) was monotosylated by the reaction with tosyl chloride to give **18** in moderate yield [29]. Nucleophilic substitution of the sulfonate against an azide then furnished **19** [30] which could by peracetylated into **20** [31] using acetic acid anhydride both in decent yields. Staudinger reduction of the azide group of **20** then afforded the desired monoaminated cyclodextrin **21** (Scheme 4).

**Scheme 5:**
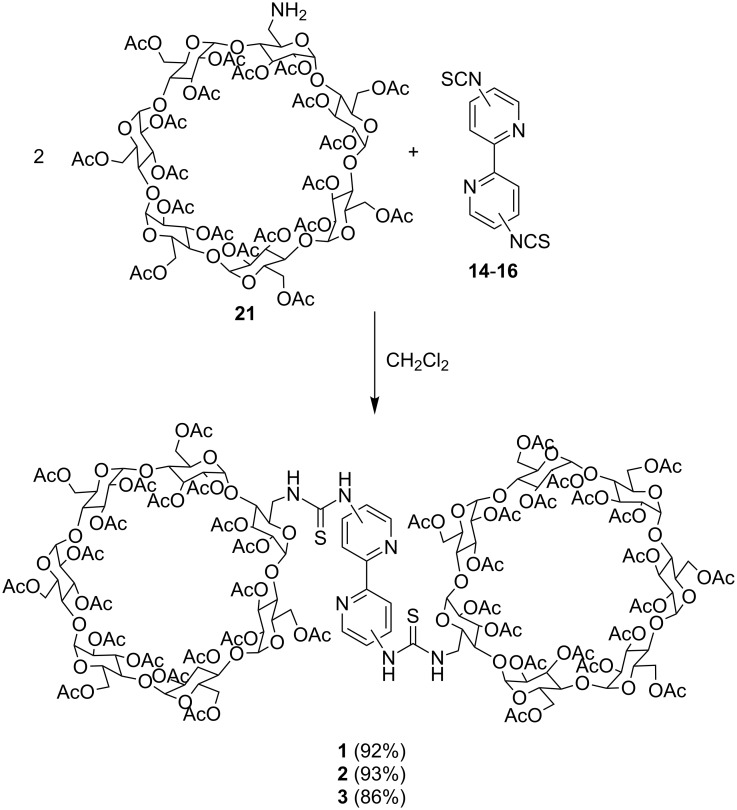
Synthesis of receptors **1–3**.

Finally, nucleophilic addition of **21** to the diisothiocyanato-2,2’-bipyridines **14**–**16** was found to proceed smoothly in dry dichloromethane at room temperature to furnish the desired bis(cyclodextrin)-functionalised 2,2’-bipyridines **1**, **2**, and **3** in very good yields of 92, 93, and 86% yield, respectively (Scheme 5).

### Metal coordination – effector binding

With our three cyclodextrin-functionalised bipyridines in hands, we then examined their coordination behaviour towards different transition metal ions (or their complexes) to see whether these could act as effectors. In our previous work, silver(I) and copper(I) salts as well as pentacarbonylrhenium(I) chloride proved to be good effectors for our allosteric receptors [20–22]. Hence, we also started with these here. Unfortunately, silver(I) ions turned out to be not effective in this case. Although, they seem to form complexes with compounds **1**–**3**, we did not observe any of the characteristic shifts of the signals assigned to the protons of the bipyridine moiety that would indicate successful binding of the silver(I) ions to the nitrogen atoms. Thus, we conclude that the soft silver(I) ions rather bind to one of the sulfur atoms of the thiourea groups. This, however, does not cause the necessary conformational change of the bipyridine structure to potentially affect the binding of another substrate.

**Figure 1:**
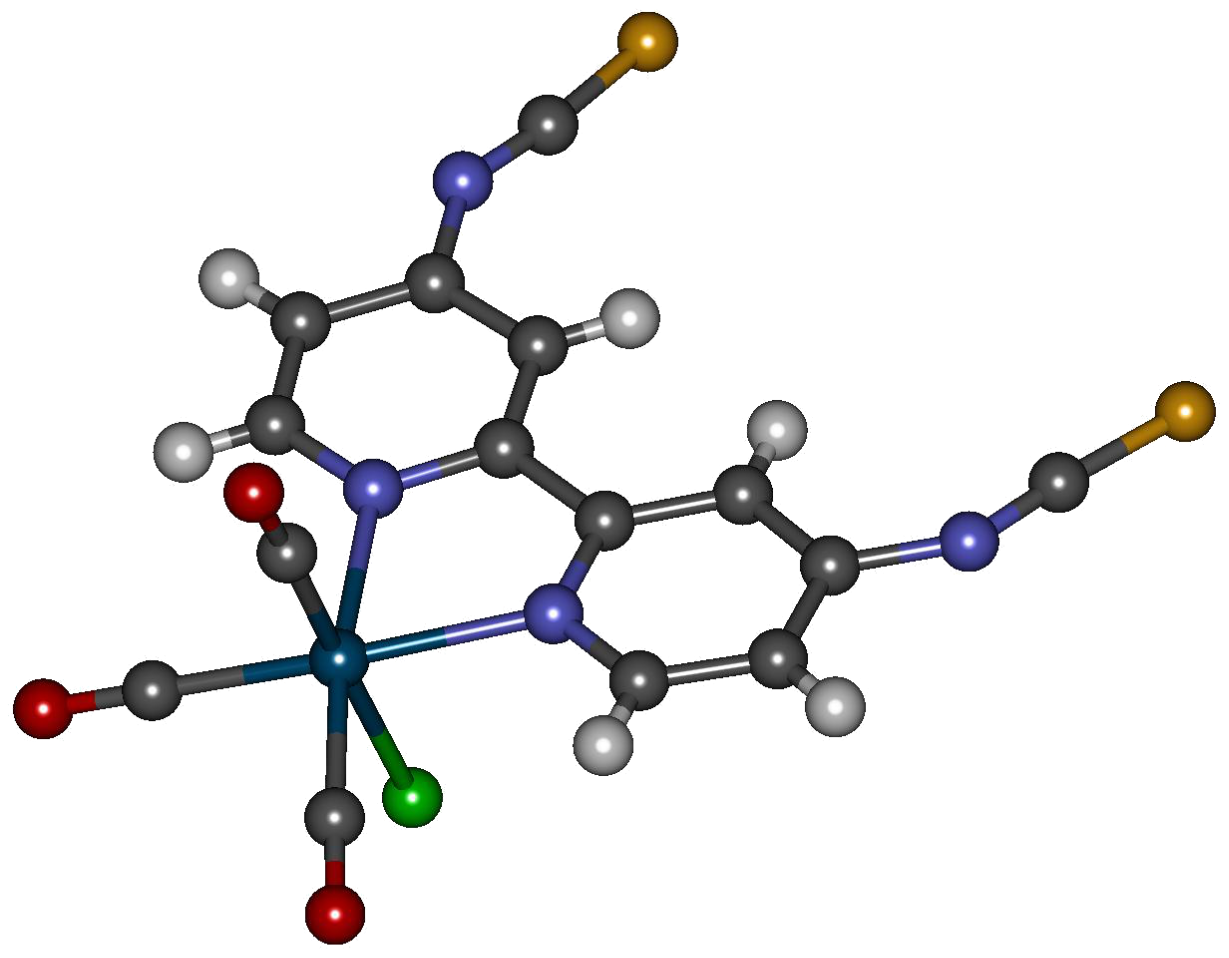
X-ray crystal structure analysis of [(CO)_3_Re(**14**)Cl] (colour code: petrol: rhenium, grey: carbon, red: oxygen, blue: nitrogen, yellow: sulfur, white: hydrogen).

Rhenium(I), on the other hand, obviously prefers coordination to nitrogen compared to sulfur as we could demonstrate by coordinating 4,4’-dithioisocyanato-2,2’-bipyridine (**14**) to it. Figure 1 shows the molecular structure of this [(CO)_3_Re(**14**)Cl] complex obtained by X-ray diffraction analysis.

Unfortunately, we were neither able to coordinate rhenium(I) to our bipyridines **1**–**3** nor did we succeed to prepare the rhenium complex [(CO)_3_Re(**1**)Cl] by using [(CO)_3_Re(**14**)Cl] and reacting it with aminocyclodextrin **21**.

**Figure 2:**
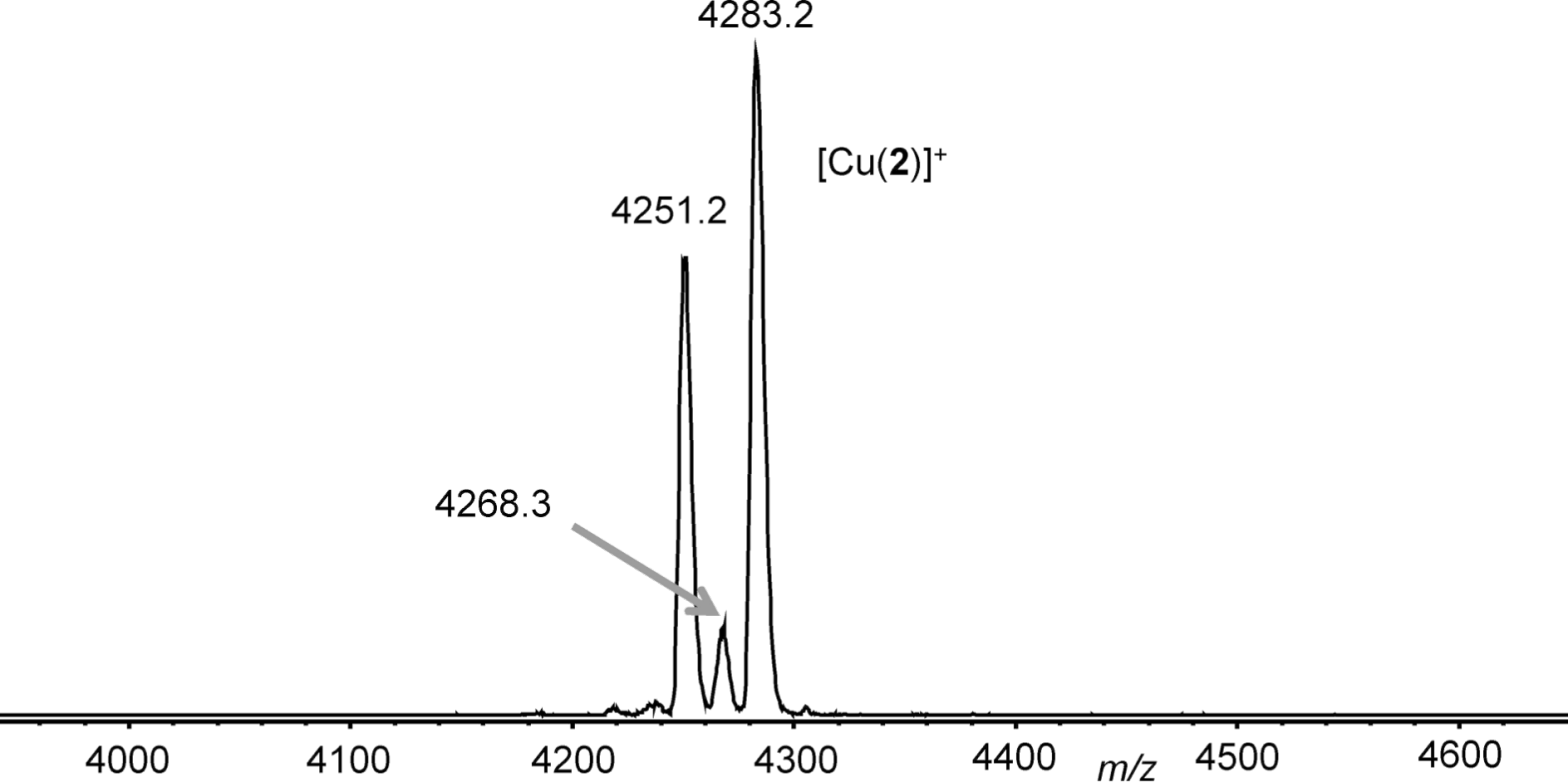
MALDI mass spectrum (sample prepared from a 1:1 mixture of CuPF_6_ and **2** in benzene/acetonitrile (1:1) using DCTB (*trans*-2-[3-(4-*tert*-butylphenyl)-2-methyl-2-propenylidene]malononitrile) as matrix).

Next, we tried copper(I) ions as effectors. Copper(I) ions indeed form complexes very fast. However, the complexes of **1** and **3** are obviously prone to rapid oxidation to copper(II) species. These are paramagnetic complexes which cannot be analysed by NMR spectroscopy. Since this technique would be our preferred analytical tool to study the host–guest chemistry of these systems in the future, however, we did not investigate these further. Interestingly, the copper(I) complexes of **2** were much less sensitive to oxidation. This might be a consequence of the steric crowding caused by the 6,6’-disubstitution of the bipyridine which was found to give well-defined 1:1 [Cu(**2**)]^+^ complexes in this case (Figure 2).

**Figure 3:**
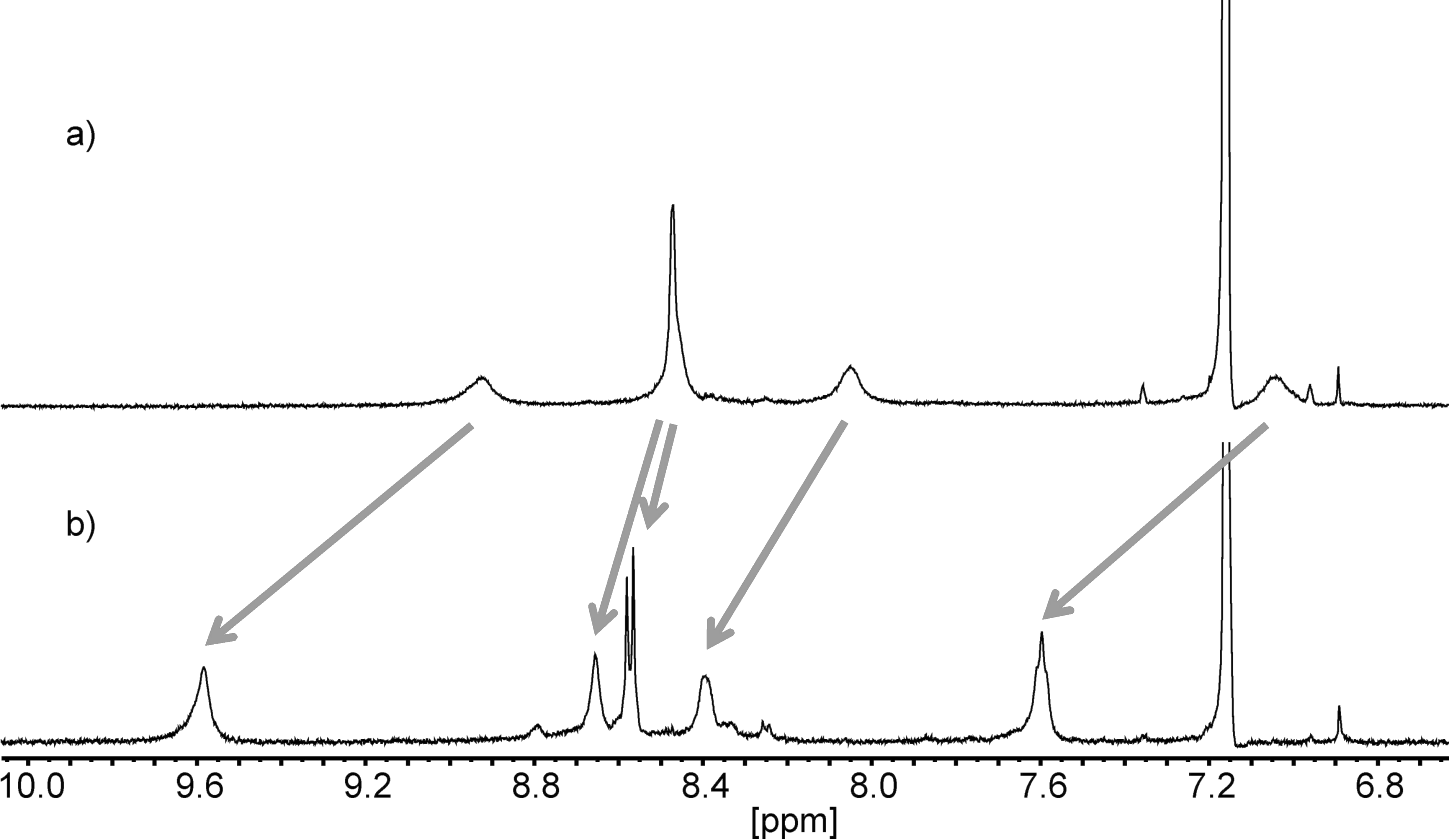
Aromatic region of the ^1^H NMR spectra (400.1 MHz, 293 K, benzene-*d*_6_/acetonitrile-*d*_3_ 1:1) of a) **1** and b) [Zn(**1**)_2_](OTf)_2_. Arrows indicate coordination-induced shifts of the individual signals.

We then turned our attention to zinc(II) ions. These proved to be capable to act as effectors in the desired manner. We observed sharp signals in the NMR when we mixed the Zn(BF_4_)_2_ and ligands **1** (Figure 3) and **3** in a 1:2 ratio.

However, in the latter case, the expected [Zn(**3**)_2_] or [Zn(**3**)] complexes could not be identified by mass spectrometry. Hence, we hesitate to claim that zinc(II) ions are reliable effectors for ligand **3**. In the case of **1**, however, we were able to find signals corresponding to the expected well defined [Zn(**1**)_2_] complexes which, together with the NMR data, proof that zinc(II) ions are suitable to act as an effector for **1**.

**Figure 4:**
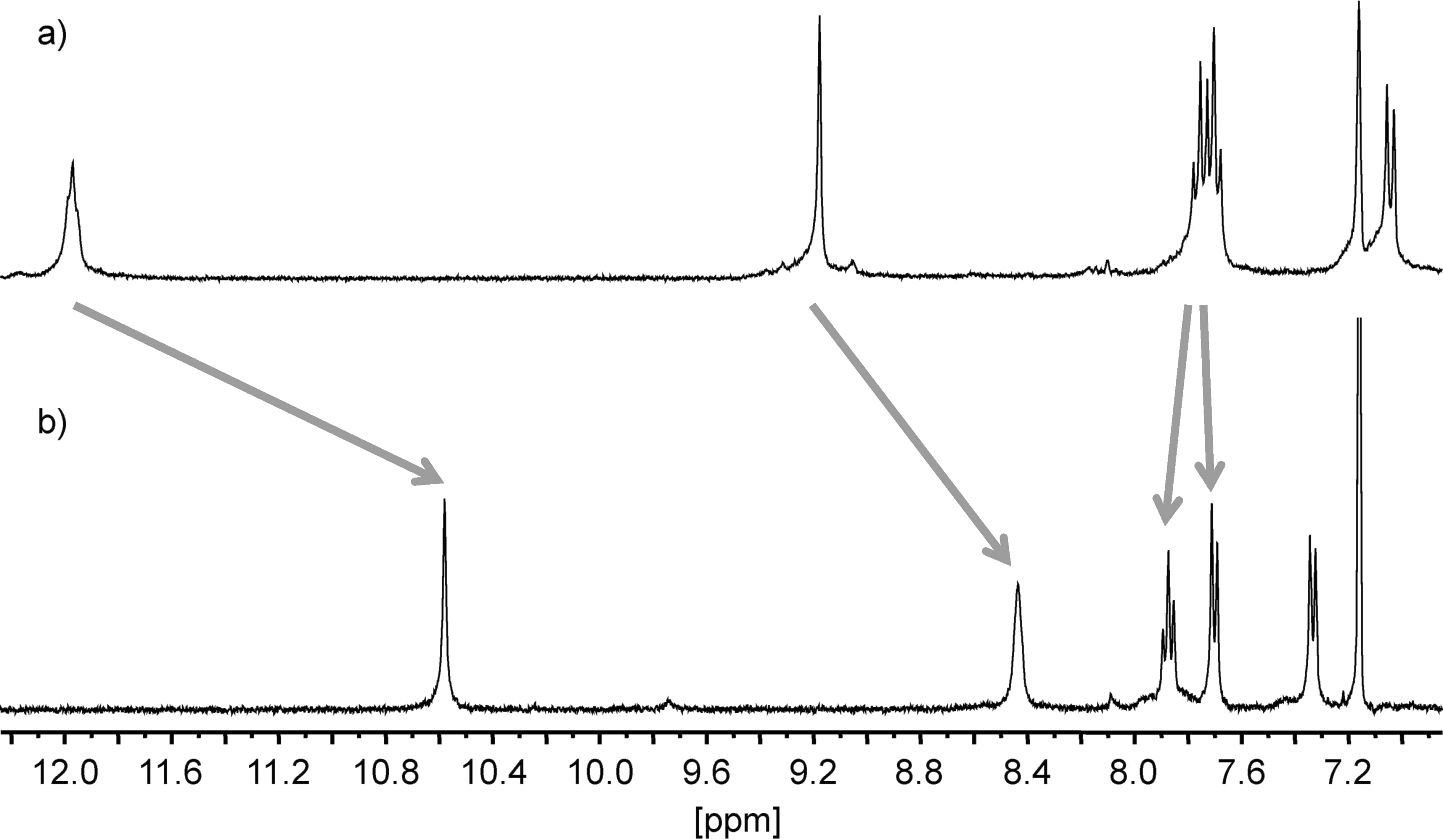
Aromatic region of the ^1^H NMR spectra (400.1 MHz, 293 K, benzene-*d*_6_/acetonitrile-*d*_3_ 1:1) of a) **2** and b) [Zn(**2**)](OTf)_2_. Arrows indicate coordination-induced shifts of the individual signals.

Changing the substitution pattern to 6,6’ like in ligand **2**, however, makes it impossible to form a 1:2 complex due to the steric crowding around the metal binding site. In this case we again observed only a 1:1 complex which probably coordinates solvent molecules to saturate the metal ion’s coordination sphere as we did already in the case of copper(I) ions (Figure 4). Thus, copper(I) and zinc(II) ions can both act as effectors for **2**.

**Scheme 6:**
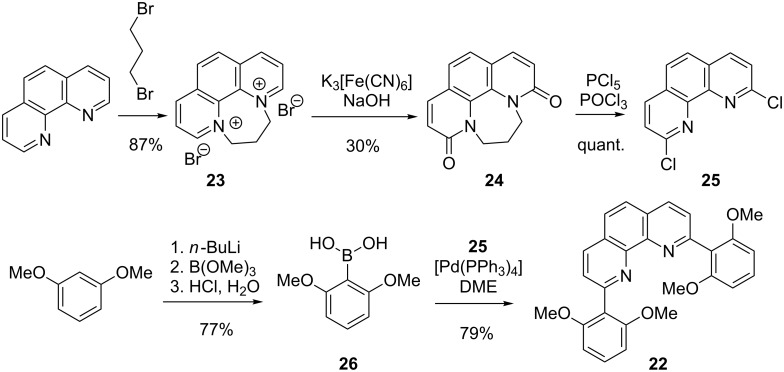
Synthesis of ligand **22**.

In order to investigate the metal complexes’ host–guest chemistry at a later stage in a quantitative manner, however, a 1:2 metal-to-ligand stoichiometry like in the [Zn(**1**)_2_]-complexes is not very easy to study because one has to deal with a very complicated equilibrium between 1:2:2, 1:2:1, 1:2:0 and even more complicated metal-to-ligand-to-substrate assemblies which would be very difficult to handle. Thus, we wanted to restrict our systems to a 1:1 stoichiometry of metal-to-ligand complexes also with ligands **1** and **3**. This can be achieved by blocking parts of the coordination sphere of a metal ion by a kinetically and thermodynamically stable binding ligand. In this way one can make sure that just one single other chelating ligand like a 2,2’-bipyridine can be bound to this metal complex fragment. Sterically hindered 1,10-phenanthrolines and their copper(I) complexes have been proven to be perfectly suited for this purpose [21,32–35]. Thus, we chose to prepare 2,9-bis[2,6-dimethoxyphenyl]-1,10-phenanthroline (**22**) as such a sterically congested ligand (Scheme 6) [36].

**22** was synthesised starting from 1,10-phenanthroline via *N*,*N*'-dialkylation to dibromide **23** in very good yield [37]. Oxidation of **23** with potassium hexacyanoferrate then afforded diamide **24** in moderate yield [38]. Its subsequent chlorination with a mixture of phosphorous pentachloride and phosphoryl chloride furnished dichloride **25** in quantitative yield [38]. Finally, two-fold Suzuki cross-coupling of **25** with 2,6-dimethoxyphenylboronic acid (**26**) derived from 1,3-dimethoxybenzene via lithiation and borylation [39] afforded **22** in very good yield [40–42].

**Figure 5:**
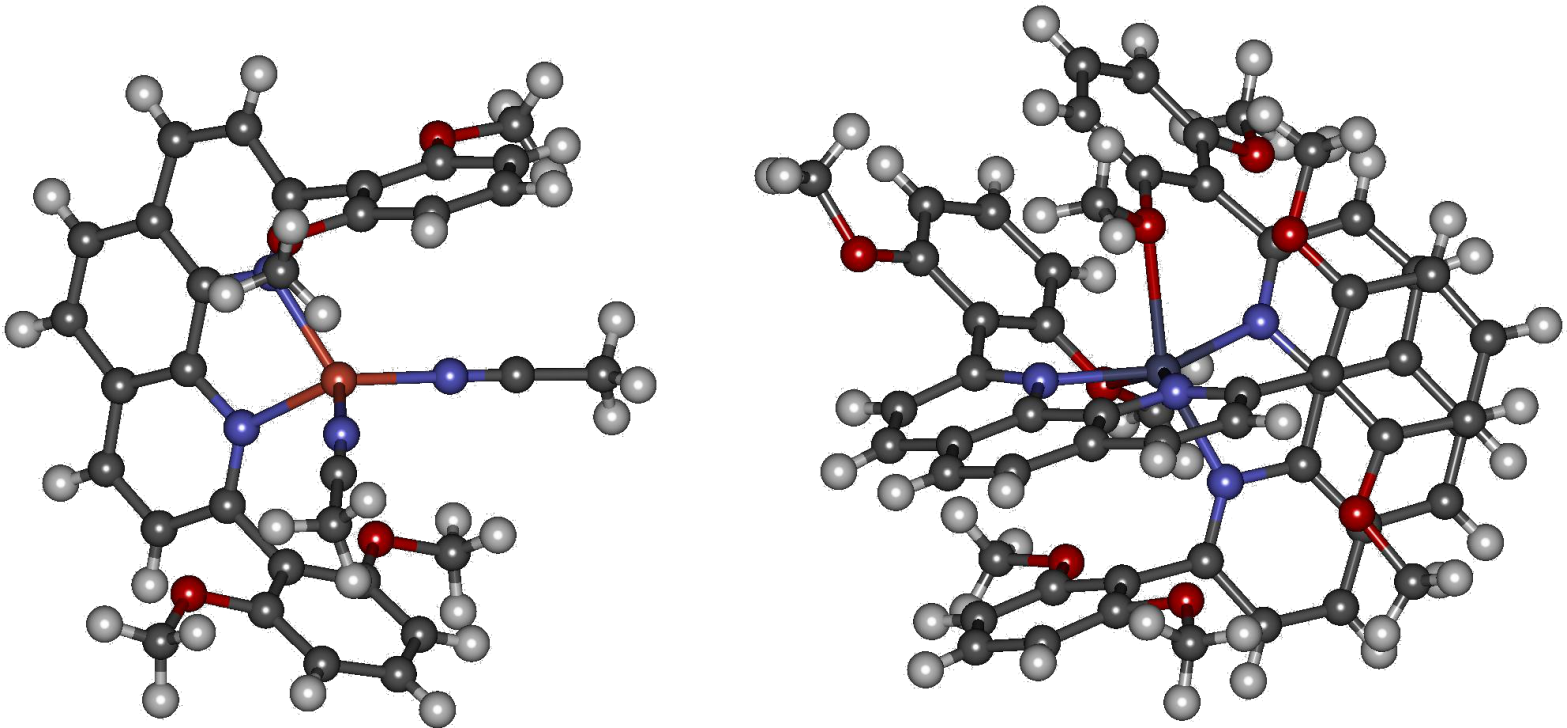
X-ray crystal structure analysis of [Cu(H_3_CCN)_2_(**22**)]BF_4_ and [Zn(**22**)_2_](OTf)_2_ (counterions are omitted, colour code: orange: copper, petrol: zinc, grey: carbon, red: oxygen, blue: nitrogen, white: hydrogen).

**22** forms the 1:1 complex [Cu(**22**)([H_3_CCN)_2_]BF_4_ upon coordination to copper(I) ions. Zinc(II) ions were found to form the dimeric complex [Zn(**22**)_2_](OTf)_2_ with an excess of **22** if no other ligand is available, as described before (Figure 5) [43].

Unfortunately, the strategy to prepare 1:1 complexes [Zn(**22**)]^2+^ and [Cu(**22**)]^+^ first and then let them react with **3** did not result in the formation of the desired heteroleptic complexes. Obviously, the complex fragments are just too large to fit into the sterically congested metal binding site of **3**. Hence, we have to conclude that, unfortunately, we have not succeeded in finding a suitable effector for this ligand yet.

**Figure 6:**
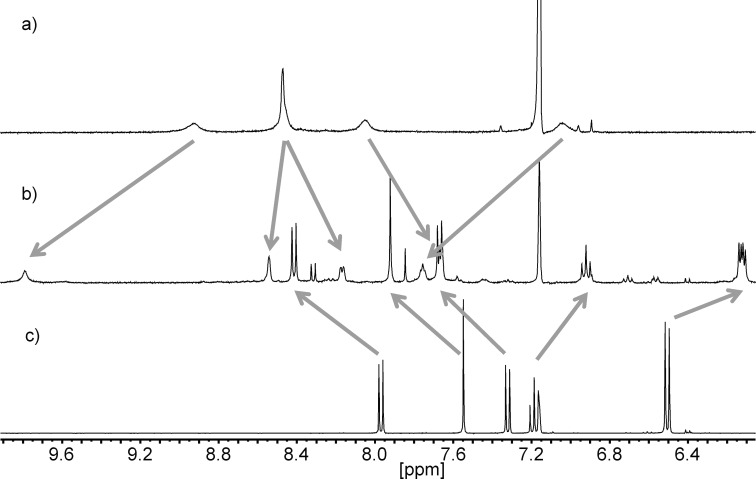
Aromatic region of the ^1^H NMR spectra (400.1 MHz, 293 K, benzene-*d*_6_/acetonitrile-*d*_3_ 1:1) of a) **1**, b) [Zn(**22**)(**1**)](OTf)_2_, and c) **22**. Arrows indicate signal shifts upon formation of the heteroleptic complex.

**Figure 7:**
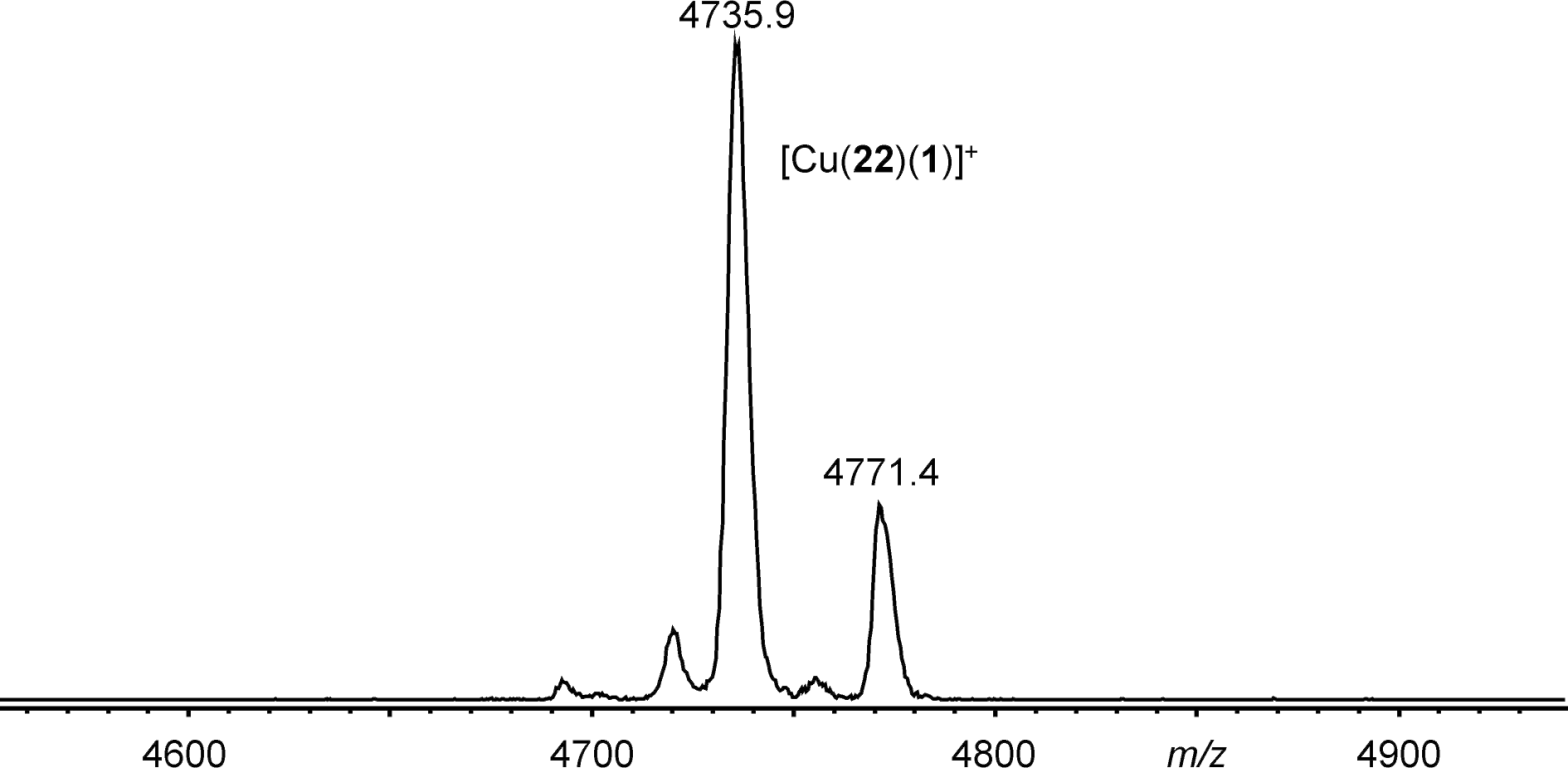
MALDI–TOF mass spectrum (sample prepared from of a 1:1:1 mixture of CuPF_6_, **22**, and **1** in benzene/acetonitrile (1:1) using DCTB as matrix).

Mixing of preformed complexes [Zn(**22**)]^2+^ and [Cu(**22**)]^+^ with **1** in a 1:1 ratio, however, afforded the desired heteroleptic complexes [Cu(**22**)(**1**)]PF_6_, and [Zn(**22**)(**1**)](OTf)_2_ as evidenced by MALDI mass spectrometry and NMR spectroscopy (Figure 6 and Figure 7).

Hence, zinc(II) ions or their complexes with a single, sterically demanding phenanthroline ligand like **22** as well as a similar [Cu(**22**)]^+^ complex were all found to be good effectors for ligand **1** forming either 1:2 or 1:1 effector:ligand complexes.

## Conclusion

We have synthesised three new β-cyclodextrin-functionalised 2,2’-bipyridines and their complexation behaviour towards several metal salts or their complexes was investigated. Among those (pentacarbonyl)rhenium chloride and silver(I) ions proved to be ineffective to cause the desired long-range conformational changes. Unfortunately, we have also not yet succeeded in finding a suitable metal ion or a complex fragment that can efficiently bind to 4,6'-disubstituted bipyridine **3**, and hence, act as an effector to cause switching from the closed "*on*"-state to the open "*off*"-state. The sterically crowded 6,6'-disubtituted bipyridine **2**, however, readily formed 1:1 complexes with both zinc(II) and copper(I) ions, and hence, can be switched between a closed "*on*"-state to an open "*off*"-state. Zinc(II) ions do also form the expected dimeric complexes with 4,4'-disubstituted bipyridine **1**. However, such a 1:2 effector–bipyridine ratio could lead to a difficult-to-handle host–guest chemistry when it comes to binding of additional guest molecules due to very complicated equilibria between 1:2:2, 1:2:1, 1:2:0 and even more complicated metal-to-ligand-to-substrate assemblies. Zinc(II) or copper(I) phenanthroline complexes with sterically very demanding phenanthrolines were found to be effective to achieve a 1:1 effector–receptor stoichiometry. This makes these ligands promising candidates that could act as potential allosteric receptors whose recognition behaviour can be largely influenced by those effectors. We are currently evaluating the host–guest chemistry of these compounds and their metal complexes which will be reported in near future.

## Supporting Information

Experimental data of all new compounds, NMR and ESI mass spectra of compounds **1**–**3**, **14**, **21**, and **22** and their metal complexes.

File 1 Experimental data, NMR and ESI mass spectra.
