# Supplementary material for: Towards allosteric receptors – synthesis of β-cyclodextrin-functionalised 2,2’-bipyridines and their metal complexes
Source: Beilstein J Org Chem. 2014 Apr 9;10:814–24. doi: 10.3762/bjoc.10.77 (PMC3999858; doi:10.3762/bjoc.10.77)
Supplement: File 1 — Experimental data, NMR and ESI mass spectra. [file Beilstein_J_Org_Chem-10-814-s001.pdf]

Supporting Information

for

Towards allosteric receptors – synthesis of β-cyclodextrin-functionalised 2,2'-bipyridines and their metal complexes

Christopher Kremer[1], Gregor Schnakenburg[2] and Arne Lützen*[1]

Address: [1]University of Bonn, Kekulé-Institute of Organic Chemistry and Biochemistry, Gerhard-Domagk-Str. 1, D-53121 Bonn, Germany and [2]University of Bonn, Institute of Inorganic Chemistry, Gerhard-Domagk-Str. 1, D-53121 Bonn, Germany

Email: Arne Lützen - arne.luetzen@uni-bonn.de

* Corresponding author

Experimental data, NMR and ESI mass spectra

- Experimental part
- NMR spectra of compounds **1**, **2**, **3**, **21**, **22** and **6** and the metal complexes of **1**, **2**, **3**, **14**, and **22**.
- Mass spectra of metal complexes of **1**, **2**, **14**, and **22**.

Experimental

General remarks: All solvents were distilled and dried prior to use according to standard procedures. All syntheses with air- and moisture-sensitive compounds were performed using Schlenk techniques under argon atmosphere. Column chromatography was performed on silica gel 60 M (0.04–0.063 mm) from Macherey-Nagel. All solvents used as eluents for column chromatography were distilled prior to use. ^{1}H and ^{13}C NMR spectra were recorded at 293 K on a Bruker AM 300 (^{1}H: 300.1 MHz, ^{13}C: 75.5 MHz) or a Bruker AM 400 (^{1}H: 400.1 MHz, ^{13}C: 100.6 MHz). ^{1}H NMR chemical shifts are reported on the δ scale (ppm) relative to residual non-deuterated solvent as internal standard. ^{13}C NMR chemical shifts are given as δ values (ppm) relative to signals of the deuterated solvent as internal standards. Mass spectra were taken on a Bruker autoflex II TOF/TOF (MALDI) or a Bruker micrOTOF-Q (ESI, Hi-Res-ESI). Elemental analyses were carried out on a Heraeus Vario EL. Chemicals and reagents (except for the solvents) obtained from commercial sources were used as received. The following compounds were prepared according to published procedures: pyrrole-substituted 2-halogenopyridines **6** and **7**,[1] bis(pyrrole)-substituted 2,2'-bipyridines **8** and **9**[1] as well as **10**[2], diamino-2,2'-bipyridines **11**–**13**[1], diisothiocyanato-2,2'-bipyridines **14**–**16**[3], 6^{A}-O-p-toluenesulfonyl-β-cyclodextrin (**18**)[4], 6^{A}-azido-6^{A}-deoxy-β-cyclodextrin (**19**)[5], 2^{A},2^{B},2^{C},2^{D},2^{E},2^{F},2^{G},3^{A},3^{B},3^{C},3^{D},3^{E},3^{F},3^{G},6^{B},6^{C},6^{D},6^{E},6^{F},6^{G}-icosa-O-acetyl-6^{A}-azido-

1 Kiehne U.; Bunzen, J.; Staats, H.; Lützen, A. *Synthesis* **2007**, 1061–1069.
2 Hapke, M.; Staats, H.; Wallmann, I.; Lützen, A. *Synthesis* **2007**, 2711–2719.
3 Kremer, C.; Lützen, A. *Synthesis* **2011**, 210–212.
4 Lee, T.; Lim, J.; Chung, I.; Kim, I.; Ha, C.-S. *Macromol. Res.* **2010**, *18*, 120–128.
5 Hbaieb, S.; Kalfat, R.; Chevalier, Y.; Amdouni, N.; Parrot-Lopez, H. *Mat. Sci. Eng. C* **2008**, *28*, 697–704.

6^A-desoxy-β-cyclodextrin (**20**)[6], 6,7-dihydro-5*H*-[1,4]di-azepino[1,2,3,4-*l,m,n*][1,10]-phenanthroline-4,8-diium dibromide (**23**)[7], 3,6,7,9-tetrahydro-5*H*-[1,4]diazepino-[1,2,3,4-*l,m,n*][1,10]-phenanthroline-3,9-dione (**24**)[7], 2,9-dichloro-1,10-phenanthroline (**25**)[8], 2,6-dimethoxyphenylboronic acid (**26**)[9], and 2,9-bis(2,6-dimethoxyphenyl)-1,10-phenanthroline (**22**)[10-12].

$2^A,2^B,2^C,2^D,2^E,2^F,2^G,3^A,3^B,3^C,3^D,3^E,3^F,3^G,6^B,6^C,6^D,6^E,6^F,6^G$-Icosa-O-acetyl-$6^A$-amino-$6^A$-desoxy-β-cyclodextrin (**21**): 3.92 g (1.96 mmol) of peracetylated azidocyclodextrin **20** were dissolved in 12 mL of dry acetone and 1.03 g (3.95 mmol) PPh_3 were added. The reaction mixture was stirred for two hours at rt, followed by the addition of 1 mL of water. After 30 minutes of reflux, the solvents were evaporated, the crude product was dissolved in dichloromethane and washed with water. After drying with $MgSO_4$, the solvents were evaporated. Further purification could be achieved by column chromatography on silica gel (eluent: dichloromethane/EtOH 96:4 + 0.5% NEt_3, R_f = 0.32) to give 2.34 g (1.18 mmol, 60%) of the desired product as a white solid.

Mp (°C): 150°C. ^{1}H NMR (300.1 MHz, $CDCl_3$): δ [ppm] = 5.23-5.40 (m, 7H, H-3^{A-G}); 5.17 (d, ^3J = 3.8 Hz, 1H, H-1^A); 5.03-5.35 (m, 6H, H-1^{B-G}); 4.72-4.86 (m, 7H, H-2^{A-G}), 4.48-4.62 (m, 6H, H-6^{B-G}); 4.19-4.35 (m, 6H, H-6^{B-G}); 4.02-4.19 (m, 7H, H-5^{A-G}); 3.82-3.98 (m, 2H, H-6^A), 3.64-3.77 (m, 7H, H-4^{A-G}); 1.95-2.20 (m, 60H, -COO*Me*). ^{13}C NMR (75.5 MHz, $CDCl_3$): δ [ppm] = 169.38-170.77 (-*C*OOMe); 96.79-98.42 (C-1^{A-G}); 76.51-77.40 (C-4^{A-G}); 69.38-71.24 (C-2^{A-G}, C-3^{A-G}, C-5^{A-G}); 62.44-62.78

6 Sallas, F.; Marsura, A.; Petot, V. *Helv. Chim. Acta* **1998**, *81*, 632–645.
7 Yamada, M.; Nakamura, Y.; Kuroda, S.; Shimano, I. *Bull. Chem. Soc. Jpn.* **1990**, 63, 2710–2712.
8 Frey, J.; Kraus, T.; Heitz, V.; Sauvage, J.-P. *Chem. Eur. J.* **2007**, *13*, 7584–7594.
9 Nishimura, N.; Yoza, K.; Kobayashi, K. *J. Am. Chem. Soc.* **2010**, *132*, 777–790.
10 Lüning, U.; Abbass, M.; Fahrenkrug, F. *Eur. J. Org. Chem.* **2002**, *19*, 3294– 3303.
11 Cacciapaglia, R.; Di Stefano, S.; Fahrenkrug, F.; Lüning, U.; Mandolini, L. *J. Phys. Org. Chem.* **2004**, *17*, 350–355.
12 Lüning, U.; Fahrenkrug, F. *Eur. J. Org. Chem.* **2004**, 3119–3127.

(C-6^{B-G}); 41.59 (C-6^{A}); 20.77 (-COO*Me*). MS (ESI (+)): *m/z* = 1974.4 ([(**7**)+H]$^{+}$); 999.2 ([(**7**)+H+Na]$^{2+}$). Elemental analysis: calcd. for $C_{82}H_{111}NO_{54}$ (%): C, 49.87; H, 5.67; N, 0.71; found (%): C, 49.53; H, 5.90; N, 0.89.

_N_,_N_''-(2,2'-Bipyridine)-4,4'-diylbis(_N_'-2^{A},2^{B},2^{C},2^{D},2^{E},2^{F},2^{G},3^{A},3^{B},3^{C},3^{D},3^{E},3^{F},3^{G}, 6^{B},6^{C},6^{D},6^{E},6^{F},6^{G}-icosa-O-acetyl-6^{A}-desoxy-β-cyclodextrin-6^{A}-yl)thiourea) (1)

0.05 g (0.2 mmol) of 4,4'-diisothiocyanato-2,2'-bipyridine (**14**) and 0.9 g (0.46 mmol, 2.3 equiv) of 2^{A},2^{B},2^{C},2^{D},2^{E},2^{F},2^{G},3^{A},3^{B},3^{C},3^{D},3^{E},3^{F},3^{G},6^{B},6^{C},6^{D},6^{E},6^{F},6^{G}-icosa-O-acetyl-6^{A}-amino-6^{A}-desoxy-β-cyclodextrin (**21**) were dissolved in 20 mL of dry dichloromethane and stirred for 24 hours at rt, followed by evaporation of the solvent. Column chromatography on silica gel (eluent: dichloromethane:EtOH 96:4, R_f = 0.32) gave 0.77 g (0.18 mmol, 92%) of the desired product as a slightly off-white amorphous solid.

^{1}H NMR (300.1 MHz, $CDCl_3$): δ [ppm] = 8.87 (b, 2H, NH); 8.46 (d, ^{3}J = 5.7 Hz, 2H, H-6_{Bipy}); 8.13 (s, 2H, H-3_{Bipy}); 7.97 (b, 2H, H-5_{Bipy}); 6.89 (b, 2H, NH); 5.22-5.45 (m, 14H, H-3^{A-G}); 4.96-5.24 (m, 14H, H-1^{A-G}); 4.69-4.93 (m, 14H, H-2^{A-G}), 3.93-4.66 (m, 38H, H-5^{A-G}, H-6^{B-G}); 3.61-3.88 (m, 18H, H-6^{A}; H-4^{A-G}); 1.91-2.28 (m, 120H, -COO*Me*). ^{13}C NMR (75.5 MHz, $CDCl_3$): δ [ppm] = 180.5 (C=S); 169.4-171.3 (-*C*OOMe); 155.9 (C-4_{Bipy}); 150.0 (C-2_{Bipy}); 147.2 (C-6_{Bipy}); 114.6 (C-5_{Bipy}); 111.8 (C-3_{Bipy}); 96.3-98.1 (C-1^{A-G}); 76.5-77.4 (C-4^{A-G}); 69.5-71.8 (C-2^{A-G}, C-3^{A-G}, C-5^{A-G}); 62.4-63.1 (C-6^{A-G}); 20.6 (-COO*Me*). MS (MALDI-TOF): *m/z* = 4219.8 ([(**1**)+H]$^{+}$). Elemental analysis: calcd. for $C_{176}H_{228}N_6O_{108}S_2 \cdot 7\ CH_2Cl_2$ (%): C, 45.66; H, 5.07; N, 1.75; S: 1.33; found (%): C, 45.82; H, 5.12; N, 1.85; S, 1.82.

***N*,*N*''-(2,2'-Bipyridine)-6,6'-diylbis(*N*'-$2^A,2^B,2^C,2^D,2^E,2^F,2^G,3^A,3^B,3^C,3^D,3^E,3^F,3^G$, $6^B,6^C,6^D,6^E,6^F,6^G$-icosa-O-acetyl-6^A-desoxy-β-cyclodextrin-6^A-yl)thiourea) (2)**

0.02 g (0.07 mmol) of 6,6'-diisothiocyanato-2,2'-bipyridine (**15**) and 0.34 g (0.17 mmol, 2.3 equiv) of $2^A,2^B,2^C,2^D,2^E,2^F,2^G,3^A,3^B,3^C,3^D,3^E,3^F,3^G,6^B,6^C,6^D,6^E,6^F,6^G$-icosa-O-acetyl-$6^A$-amino-$6^A$-desoxy-β-cyclodextrin (**21**) were dissolved in 10 mL of dry dichloromethane and stirred for 48 hours at rt, followed by evaporation of the solvent. Column chromatography on silica gel (eluent: dichloromethane:EtOH 96:4, R_f = 0.38) gave 0.29 g (0.069 mmol, 93%) of the desired product as a slightly off-white amorphous solid.

^{1}H NMR (400.1 MHz, $CDCl_3$): δ [ppm] = 11.81 (s, 2H, N-H); 8.97 (s, 2H, N-H); 7.77 (dd, ^3J = 7.9 Hz, 2H, H-4_{Bipy}); 7.59 (d, ^3J = 7.9 Hz, 2H, H-3_{Bipy}); 7.02 (d, ^3J = 7.9 Hz, 2H, H-5_{Bipy}); 5.10-5.40 (m, 14H, H-$3^{A\text{-}G}$); 4.91-5.09 (m, 14H, H-$1^{A\text{-}G}$); 4.64-4.83 (m, 14H, H-$2^{A\text{-}G}$), 3.88-4.27 (m, 38H, H-$5^{A\text{-}G}$, H-$6^{B\text{-}G}$); 3.42-3.81 (m, 18H, H-6^A; H-$4^{A\text{-}G}$); 1.82-2.52 (m, 120H, -COO*Me*). ^{13}C NMR (100.1 MHz, $CDCl_3$): δ [ppm] = 180.2 (C=S); 169.2-171.5 (-*C*OOMe); 153.0 (C-6_{Bipy}); 151.8 (C-2_{Bipy}); 139.6 (C-4_{Bipy}); 114.9 (C-3_{Bipy}); 113.4 (C-5_{Bipy}); 96.3-97.5 (C-$1^{A\text{-}G}$); 76.1-78.1 (C-$4^{A\text{-}G}$); 68.5-72.0 (C-$2^{A\text{-}G}$, C-$3^{A\text{-}G}$, C-$5^{A\text{-}G}$); 62.4-65.2 (C-$6^{A\text{-}G}$); 20.8-21.1 (-COO*Me*). MS (MALDI-TOF): *m*/*z* = 4217.9 ([(**2**)+H]$^+$). Elemental analysis: calcd. for $C_{176}H_{228}N_6O_{108}S_2 \cdot 2\ CH_2Cl_2$ (%): C, 48.70; H, 5.33; N, 1.91; S, 1.46; found (%): C, 48.39; H, 5.48; N, 1.56; S, 1.85.

***N*,*N*''-(2,2'-Bipyridine)-4,6'-diylbis(*N*'-$2^A,2^B,2^C,2^D,2^E,2^F,2^G,3^A,3^B,3^C,3^D,3^E,3^F,3^G$, $6^B,6^C,6^D,6^E,6^F,6^G$-icosa-O-acetyl-6^A-desoxy-β-cyclodextrin-6^A-yl)thiourea) (3)**

0.02 g (0.07 mmol) of 4,6'-diisothiocyanato-2,2'-bipyridine (**16**) and 0.34 g (0.17 mmol, 2.3 equiv) of $2^A,2^B,2^C,2^D,2^E,2^F,2^G,3^A,3^B,3^C,3^D,3^E,3^F,3^G,6^B,6^C,6^D,6^E,6^F,6^G$-Icosa-O-acetyl-$6^A$-amino-$6^A$-desoxy-β-cyclodextrin were dissolved in 10 mL of dry dichloromethane and stirred for 48 hours at rt, followed by evaporation of the solvent.

Column chromatography on silica gel (eluent: dichloromethane:EtOH 96:4, R_f = 0.38) gave 0.27 g (0.06 mmol, 86%) of the desired product as a slightly off-white amorphous solid.

^{1}H NMR (300.1 MHz, $CDCl_3$): δ[ppm] = 12.26 (b, 1H, N-H); 9.11 (s, 1H, H-3_{Bipy}); 8.63 (b, 1H, N-H); 8.53 (d, ^3J = 5.5 Hz, 1H, H-6_{Bipy}); 8.30 (b, 1H, N-H); 7.95 (d, ^3J = 7.9 Hz, 1H, H-3'$_{Bipy}$); 7.69 (m, 2H, H-4'$_{Bipy}$, H-5_{Bipy}); 7.09 (b, 1H, N-H); 7.00 (d, ^3J = 7.9 Hz, 1H, H-5'$_{Bipy}$); 5.22-5.40 (m, 14H, H-3^{A-G}); 4.96-5.22 (m, 14H, H-1^{A-G}); 4.65-4.93 (m, 14H, H-2^{A-G}), 4.03-4.63 (m, 38H, H-5^{A-G}, H-6^{B-G}); 3.57-3.91 (m, 18H, H-6^{A}; H-4^{A-G}); 1.75-2.23 (m, 120H, -COO*Me*). ^{13}C NMR (75.5 MHz, $CDCl_3$): δ[ppm] = 180.1 (C=S); 169.1-171.1 (-*C*OOMe); 155.3 (C-4_{Bipy}); 152.7 (C-6'$_{Bipy}$); 152.0 (C-2'$_{Bipy}$); 150.5 (C-6_{Bipy}); 147.2 (C-2_{Bipy}); 139.7 (C-4'$_{Bipy}$); 115.7 (C-3'$_{Bipy}$); 115.0 (C-5_{Bipy}); 113.0 (C-5'$_{Bipy}$); 112.2 (C-3); 95.5-97.9 (C-1^{A-G}); 75.9-78.4 (C-4^{A-G}); 68.7-72.3 (C-2^{A-G}, C-3^{A-G}, C-5^{A-G}); 62.0-63.1 (C-6^{A-G}); 20.4 (-COO*Me*). MS (ESI(+): *m/z* = 2132.5 ($[(\mathbf{3})+Na]^{2+}$). Hi-Res-MS (ESI(+)): calcd. for $[C_{176}H_{228}N_6O_{108}S_2Na_2]^+$: *m/z* = 2131.5880; found: *m/z* = 2131.5813 (Δ = 3.1 ppm). Elemental analysis: calcd for $C_{176}H_{228}N_6O_{108}S_2$ (%):C, 50.09; H, 5.45; N, 1.99; S, 1.52; found (%): C, 49.72; H, 5.69; N, 1.58; S, 1.57.

[(CO)$_3$Re(14)Cl]

0.02 g (0.07 mmol) of **14** and 0.03 mg (0.016 mmol) pentacarbonylrhenium(I) chloride were dissolved in 2 mL $CHCl_3$. The solution was stirred at 40 °C. After 7 d, when ^{1}H NMR measurements showed complete conversion, the solvent was evaporated.

^{1}H NMR (300.1 MHz, $CDCl_3$): δ [ppm] = 8.98 (d, ^3J = 6.0 Hz, 2H, H-6); 7.85 (d, ^4J = 2.1 Hz, 2H, H-3); 7.31 (dd, ^3J = 6.0 Hz, ^4J = 2.1 Hz, 2H, H-5). MS (ESI (+)): *m/z* = 605.0 ({[(CO)$_3$Re(**14**)]+2 MeOH}$^+$); 623.0 ([{(CO)$_3$Re(**14**)]+2 MeOH+H_2O}$^+$).

Hi-Res.-MS (ESI (+)): calcd. for $[C_{15}H_6N_4O_3ReS_2(CH_3OH)_2]^+$: *m/z* = 604.9950; found: *m/z* = 604.9940 (Δ = 1.7 ppm).

$[Zn(1)_2](OTf)_2$

0.7 mg (0.002 mmol) of $Zn(OTf)_2$ were in dissolved in 0.5 mL of C_6D_6/CD_3CN (1:1). 0.3515 mL of this solution were transferred into a solution of 8 mg (0.002 mmol) of **1** in 0.1985 mL of C_6D_6/CD_3CN (1:1) and stirred for 1 h at 40 °C.

^1H NMR (400.1 MHz, C_6D_6/CD_3CN): δ [ppm] = 9.58 (b, 2H, N-H); 8.66 (s, 2H, H-3_{Bipy}); 8.57 (d, ^3J = 6.1 Hz, 2H, H-6_{Bipy}); 8.39 (b, 2H, H-5_{Bipy}); 7.6 (b, 2H, N-H); 5.32-5.43 (m, 14H, H-3^{A-G}); 5.01-5.13 (m, 14H, H-1^{A-G}); 4.70-4.83 (m, 14H, H-2^{A-G}); 4.08-4.62 (m, 38H, H-5^{A-G}, H-6^{B-G}); 3.69-3.96 (m, 18H, H-6^A; H-4^{A-G}); 1.85-2.01 (m, 120H, -COO*Me*). MS (MALDI-TOF): *m/z* = 4432.4 $\{[(Zn(\mathbf{1})]OTf\}^+$; 8654.7 $\{[Zn(\mathbf{1})_2]OTf]^+$.

$[Cu(2)]PF_6$

2 mg (0.00537 mmol) of $Cu(MeCN)_4PF_6$ were dissolved in 0.5 mL of C_6D_6/CD_3CN (1:1). 0.177 mL of this solution were transferred into a solution of 8 mg (0.00186 mmol) of **2** in 0.423 mL of C_6D_6/CD_3CN (1:1). The yellow solution was stirred for 1 h at 40 °C.

^1H NMR (400.1 MHz, C_6D_6/CD_3CN): δ [ppm] = 12.04 (b, 2H, N-H); 9.79 (b, 2H, N-H); 7.91 (m, 2H, H-4_{Bipy}); 7.81 (m, 2H, H-3_{Bipy}); 7.31 (m, 2H, H-5_{Bipy}); 5.30-5.63 (m, 14H, H-3^{A-G}); 5.03-5.25 (m, 14H, H-1^{A-G}); 4.7-4.96 (m, 14H, H-2^{A-G}), 4.10-4.70 (m, 38H, H-5^{A-G}, H-6^{B-G}); 3.73-4.05 (m, 18H, H-6^A; H-4^{A-G}); 1.90-2.25 (m, 120H, -COO*Me*). MS (MALDI-TOF): *m/z* = 4283.3 ($[Cu(\mathbf{2})]^+$).

[Zn(2)](OTf)$_2$

1 mg (0.00275 mmol) of Zn(OTf)$_2$ were dissolved in 0.5 mL of C_6D_6/CD_3CN (1:1). 0.338 mL of this solution were transferred into a solution of 8 mg (0.00186 mmol) **2** in 0.162 mL of C_6D_6/CD_3CN (1:1) and stirred for 1 h at 40 °C.

^{1}H NMR (400.1 MHz, C_6D_6/CD_3CN): δ [ppm] = 10.58 (s, 2H, N-H); 8.43 (s, 2H, N-H); 7.87 (dd, ^3J = 8.1 Hz, ^3J = 8.1 Hz, 2H, H-4$_{Bipy}$); 7.70 (d, ^3J = 8.1 Hz, 2H, H-3$_{Bipy}$); 7.33 (d, ^3J = 8.1 Hz, 2H, H-5$_{Bipy}$); 5.35-5.50 (m, 14H, H-3$^{A\text{-}G}$); 4.91-5.21 (m, 14H, H-1$^{A\text{-}G}$); 4.68-4.95 (m, 14H, H-2$^{A\text{-}G}$); 4.05-4.59 (m, 42H, H-5$^{A\text{-}G}$, H-6$^{A\text{-}G}$); 3.75-3.92 (m, 14H, H-4$^{A\text{-}G}$); 1.82-2.08 (m, 120H, -COO*Me*). MS (ESI(+)): *m/z* = 1429.2 ({[Zn(**2**)]+H}$^{3+}$).

[Cu(22)]PF$_6$

5 mg (0.01341 mmol) of Cu(MeCN)$_4$PF$_6$ were dissolved in 0.5 mL of C_6D_6/CD_3CN (1:1). 0.297 mL of this solution were transferred into a solution of 3.6 mg (0.00796 mmol) of **22** in 0.5 mL of C_6D_6/CD_3CN (1:1). A yellow solution was obtained.

^{1}H NMR (400.1 MHz, C_6D_6/CD_3CN 1:1): δ [ppm] = 8.09 (d, $^3J_{3,4}$ = 8.3 Hz, 2H, H-4); 7.54 (s, 2H, H-5); 7.52 (d, $^3J_{3,4}$ = 8.3 Hz, 2H, H-3); 7.3 (dd, $^3J_{3',4'}$ = 8.4 Hz, 2H, H-4‘); 6.57 (d, $^3J_{3',4'}$ = 8.4 Hz, 4H, H-3‘); 3.43 (s, 12H, -O*Me*). MS (ESI(+)): *m/z* = 515.2 ([Cu(**22**)]$^+$). Hi-Res.-MS (ESI(+)): calcd. for $[C_{28}H_{24}CuN_2O_4]^+$: *m/z* = 515.1026; found: *m/z* = 515.1027 (Δ = 0.2 ppm).

[Zn(22)$_2$](OTf)$_2$

5 mg (0.014 mmol) of Zn(OTf)$_2$ were dissolved in 0.3 mL of C_6D_6/CD_3CN (1:1). 0.289 mL of this solution were transferred into a solution of 5 mg (0.01105 mmol) of **22** in 0.4 mL of C_6D_6/CD_3CN (1:1). The colourless solution was stirred at 40 °C for 1 h.

^{1}H NMR (400.1 MHz, C_6D_6/CD_3CN): δ [ppm] = 8.34 (d, $^3J_{3,4}$ = 8.4 Hz, 2H, H-4); 7.86 (s, 2H, H-5); 7.45 (d, $^3J_{3,4}$ = 8.4 Hz, 2H, H-3); 7.45 (dd, $^3J_{3',4'}$ = 8.4 Hz, 2H, H-4‘); 6.70

(d, $^3J_{3',4'}$ = 8.4 Hz, 4H, H-3'); 3.31 (s, 12H, -O*Me*). MS (ESI (+)): *m/z* = 258.0 ($[M+Zn]^{2+}$); 484.1 ($[Zn(\mathbf{22})_2]^{2+}$); 665.1 ($\{[Zn(\mathbf{11})]OTf\}^+$). Hi-Res.-MS (ESI (+)): calcd. for $[C_{29}H_{24}F_3N_2O_7SZn]^+$: *m/z* = 665.0542; found: *m/z* = 665.0522 (Δ = 3 ppm).

[Cu(1)(22)]PF$_6$

2 mg (0.005366 mmol) of $Cu(MeCN)_4PF_6$ were dissolved in 0.4 mL of C_6D_6/CD_3CN (1:1). 0.3295 mL of this solution were transferred to a solution of 2 mg (0.00442 mmol) of **22** in 0.4 mL of C_6D_6/CD_3CN (1:1). From this solution, 0.3129 mL were taken and added to a solution of 8 mg (0.001896 mmol) of **1** in 0.3 mL of C_6D_6/CD_3CN (1:1). The resulting solution has a deep red colour.

^{1}H NMR (400.1 MHz, C_6D_6/CD_3CN): δ [ppm] = 9.10 (b, 2H, N-H); 8.26 (b, 2H, H-3$_{Bipy}$); 8.14 (d, ^3J = 8.3 Hz, 2H, H-4$_{Phen}$); 7.85 (b, 2H, H-5$_{Bipy}$); 7.82 (d, ^3J = 5.7 Hz, 2H, H-6$_{Bipy}$); 7.60 (s, 2H, H-5$_{Phen}$); 7.52 (d, ^3J = 8.3 Hz, 2H, H-3$_{Phen}$); 7.19 (b, 2H, N-H); 6.79 (dd, ^3J = 8.4 Hz, 2H, H-4'$_{Phen}$); 6.01 (d, ^3J = 8.4 Hz, 4H, H-3'$_{Phen}$); 5.35-5.48 (m, 14H, H-3^{A-G}); 5.02-5.18 (m, 14H, H-1^{A-G}); 4.70-4.85 (m, 14H, H-2^{A-G}); 4.05-4.55 (m, 38H, H-5^{A-G}, H-6^{B-G}); 3.69-3.92 (m, 18H, H-6^{A}; H-4^{A-G}); 3.21 (s, 12H, -O*Me*); 1.85-1.98 (m, 120H, -COO*Me*). MS (MALDI-TOF): 4735.9 ($[Cu(\mathbf{1})(\mathbf{22})]^+$).

[Zn(1)(22)](OTf)$_2$

2.5 mg (0.00688 mmol) of $Zn(OTf)_2$ were dissolved in 0.6 mL C_6D_6/CD_3CN (1:1). 0.4821 mL of this solution were transferred into a solution of 2.5 mg (0.00553 mmol) of **22** in 0.6 mL C_6D_6/CD_3CN (1:1). From this solution, 1.0211 mL were taken and added to a solution of 22 mg (0.005214 mmol) of **1** in 0.3 mL of C_6D_6/CD_3CN (1:1). The colourless mixture was stirred for 1 h at 40 °C.

^{1}H NMR (400.1 MHz, C_6D_6/CD_3CN): δ [ppm] = 9.79 (b, 2H, N-H); 8.54 (s, 2H, H-3$_{Bipy}$); 8.41 (d, ^3J = 8.4 Hz, 2H, H-4$_{Phen}$); 8.17 (d, ^3J = 6.0 Hz, 2H, H-6$_{Bipy}$); 7.92 (s,

2H, H-5$_{Phen}$); 7.75 (b, 2H, N-H); 7.67 (d, ^{3}J = 8.4 Hz, 2H, H-3$_{Phen}$); 7.66 (d, ^{3}J = 6.0 Hz, 2H, H-5$_{Bipy}$); 6.92 (dd, ^{3}J = 8.4 Hz, 2H, H-4'$_{Phen}$); 6.13 (m, 4H, H-3'$_{Phen}$); 5.35-5.50 (m, 14H, H-3$^{A\text{-}G}$); 5.02-5.19 (m, 14H, H-1$^{A\text{-}G}$); 4.68-4.80 (m, 14H, H-2$^{A\text{-}G}$); 4.10-4.48 (m, 42H, H-5$^{A\text{-}G}$, H-6$^{A\text{-}G}$); 3.75-3.90 (m, 14H, H-6^{A}; H-4$^{A\text{-}G}$); 3.19 (s, 12H, -O*Me*); 1.86-1.98 (m, 120H, -COO*Me*). MS (MALDI-TOF): *m/z* = 4884.6 {[Zn(**1**)(**22**)]OTf}$^{+}$.

Crystal structure determination: The data collection was performed on a NONIUS KappaCCD diffractometer (area detector) using graphite monochromated Mo K_{α} radiation (λ = 0.71073 Å). The diffractometer was equipped with a low–temperature device (Cryostream 600er series, Oxford Cryosystems, 123(2) K). Intensities were measured by fine-slicing ω and φ-scans and corrected for background, polarization and Lorentz effects.

An empirical absorption correction was applied for all data sets according to Blessing's method.[13] The structures were solved by direct methods and refined anisotropically by the least-squares procedure implemented in the ShelX program system.[14] Hydrogen atoms were included isotropically using the riding model on the bound carbon atoms.

CCDC-974931 ([(CO)$_3$Re(**14**)Cl]), CCDC-974932 ([Cu(**22**)(H_3CCN)$_2$]PF_6), and CCDC-974933 ([Zn(**22**)$_2$](OTf)$_2$) contain the supplementary crystallographic data for this paper, which can be obtained free of charge from The Cambridge Crystallographic Data Centre via www.ccdc.cam.ac.uk/data_request/cif.

13 Blessing, R. H. *Acta Cryst.*, **1995**, *A61*, 33–38
14 Sheldrick, G. M.; *SHELXS97* and *SHELXL97*, University of Göttingen, Germany, **1997**.

Table S1: Crystallographic data for $[(CO)_3Re($**14**$)Cl]$, $[Cu($**22**$)(H_3CCN)_2]PF_6$, and $[Zn($**22**$)_2](OTf)_2$.

Parameters	$[(CO)_3Re($**14**$)Cl]$	$[Cu($**22**$)(H_3CCN)_2]PF_6$	$[Zn($**22**$)_2](OTf)_2$
formula	$C_{16}H_7Cl_4N_4O_3ReS_2$	$C_{32}H_{30}CuF_6N_4O_4P$	$C_{58}H_{48}F_6N_4O_{14}S_2Zn$
M_r	695.38	743.11	1268.49
T [K]	123(2)	123(2)	123.2(1)
crystal system	monoclinic	triclinic	triclinic
space group	P $2_1/c$	*P* -1	*P* -1
crystal dimensions [mm]	0.32 × 0.18 × 0.08	0.28 × 0.20 × 0.02	0.40 × 0.12 × 0.08
a [Å]	11.7253(2)	11.1033(8)	10.6987(2)
b [Å]	17.6132(3)	12.4397(9)	12.9294(3)
c [Å]	11.6498(2)	13.5810(10)	19.7165(4)
α [°]	90	66.770(2)	90.5060(12)
β [°]	115.0710(10)	87.495(2)	92.8121(14)
γ [°]	90	71.359(2)	101.5840(12)
V [$Å^3$]	2179.24(6)	1620.0(2)	2668.08(10)
Z	4	2	2
ρ [mg m^3]	2.119	1.518	1.579
μ [mm^{-1}]	6.285	0.799	0.634
F(000)	1320	760	1304
θ range [°]	3.00-28.00	2.66-28.00	2.16-28.00
completeness [%]	99.8	99.6	99.7
reflections measured	58475	42418	67742
unique reflections	5260/	7816	12833
(R_{int})	(0.0693)	(0.0632)	(0.0719)
data/restrains/para-meters	5260/2/271	7816/6/445	12833/101/812
GoF on F^2	1.083	1.033	1.055
final *R* indices	R1 = 0.0299	R1 = 0.0588	R1 = 0.0694
[$I > 2\sigma(I)$]	ωR2 0.0566	ωR2 = 0.1415	ωR2 = 0.2157
R indices all data	R1 = 0.0299	R1 = 0.0998	R1 = 0.1024
	ωR2 = 0.0583	ωR2 = 0.1659	ωR2 = 0.2364
largest diff. peak and hole [e $Å^3$]	1.090/ –1.507	1.250/ –1.236	3.587/–2.247

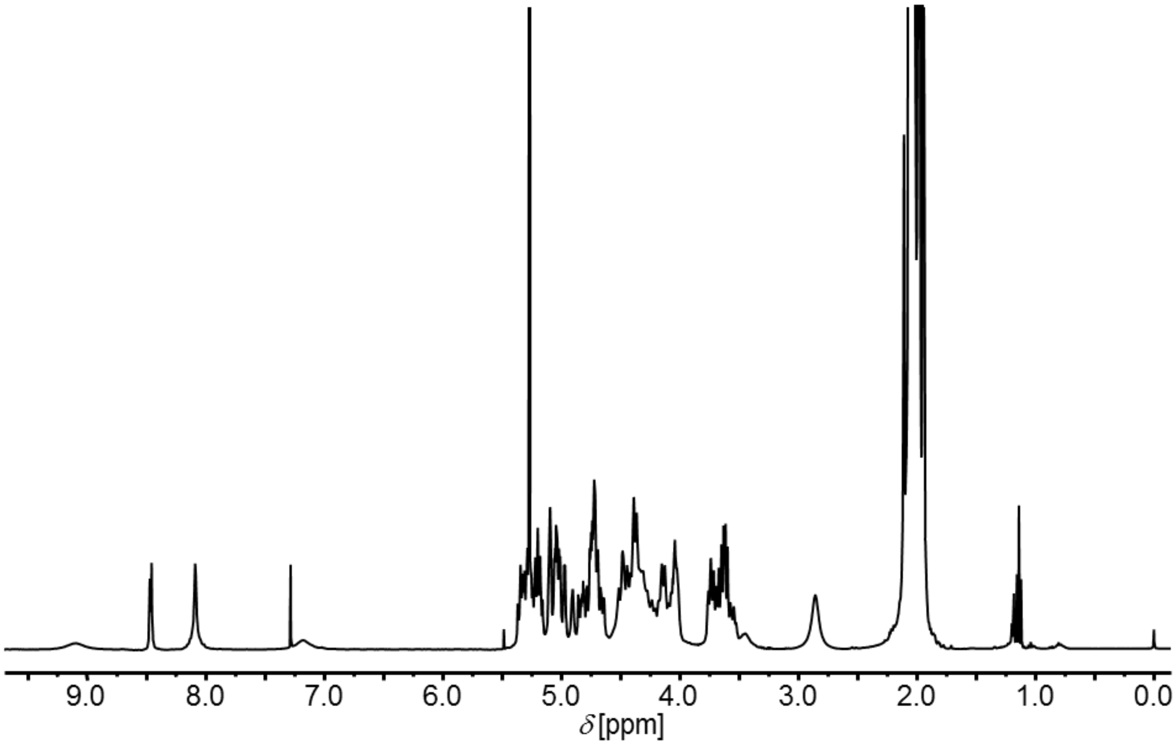

Figure S1: ^{1}H NMR spectrum (400.1 MHz, in $CDCl_3$ at 293 K) of **1**.

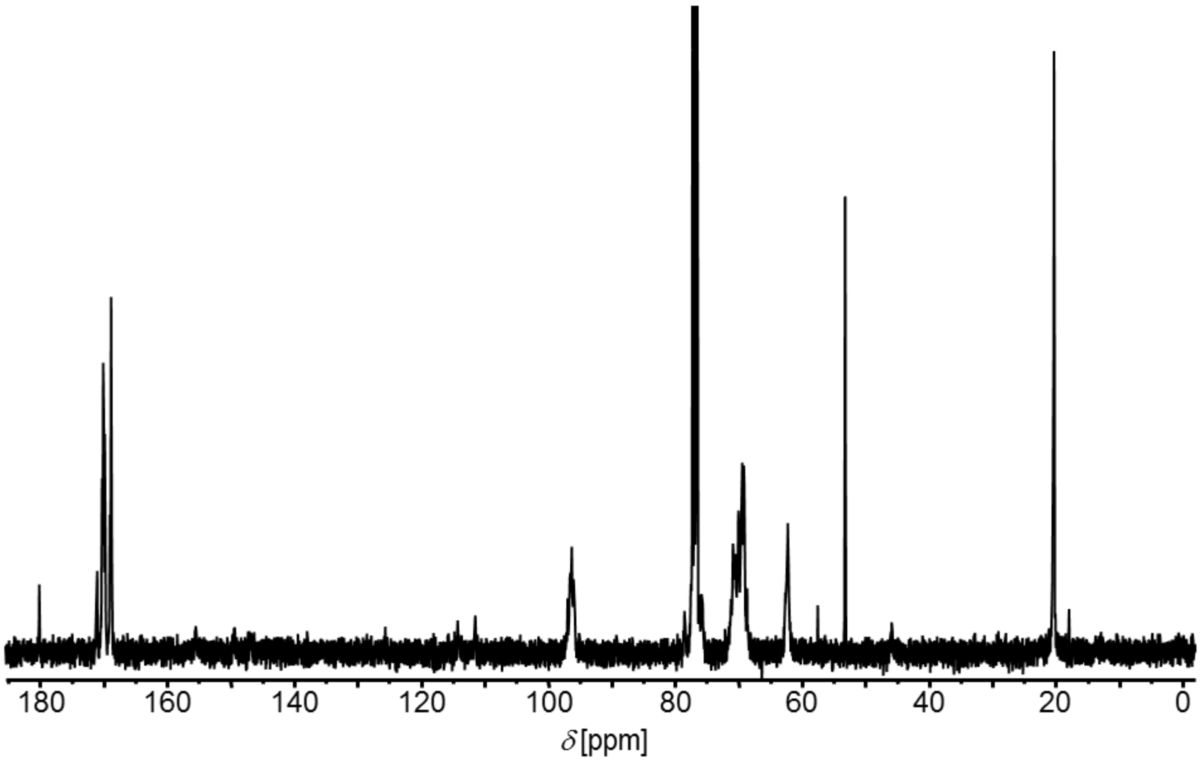

Figure S2: ^{13}C NMR spectrum (100.6 MHz, in $CDCl_3$ at 293 K) of **1**.

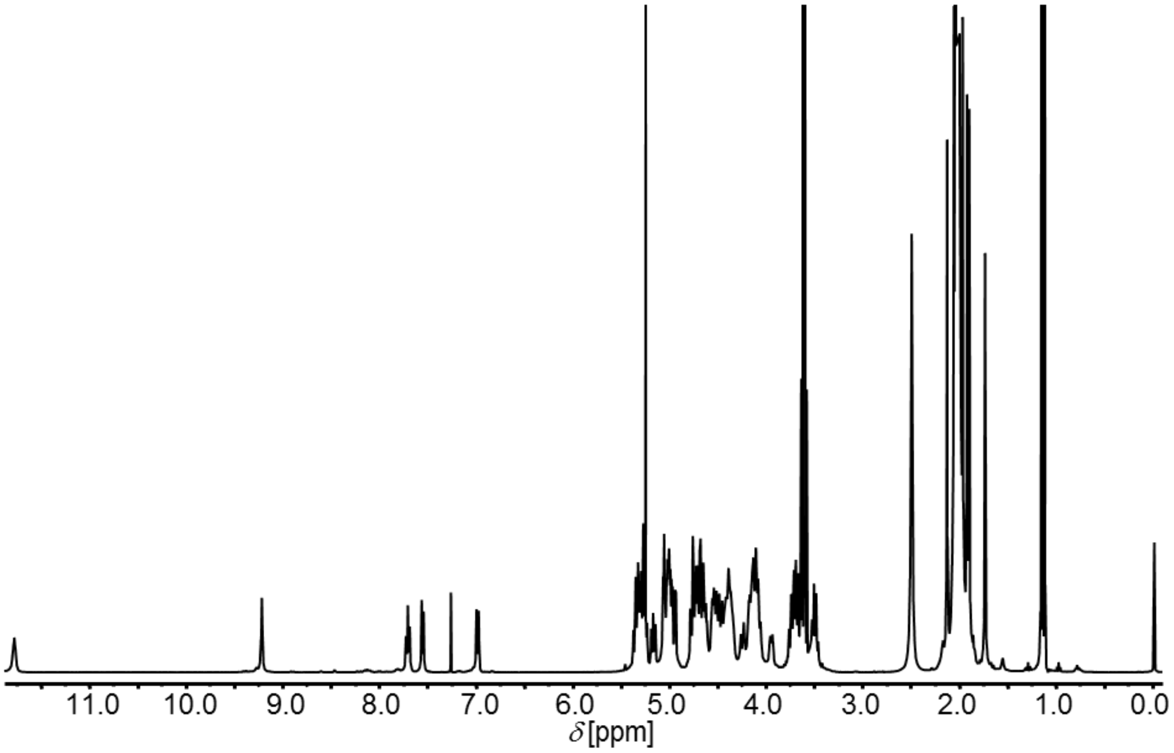

Figure S3: ^{1}H NMR spectrum (400.1 MHz, in $CDCl_3$ at 293 K) of **2**.

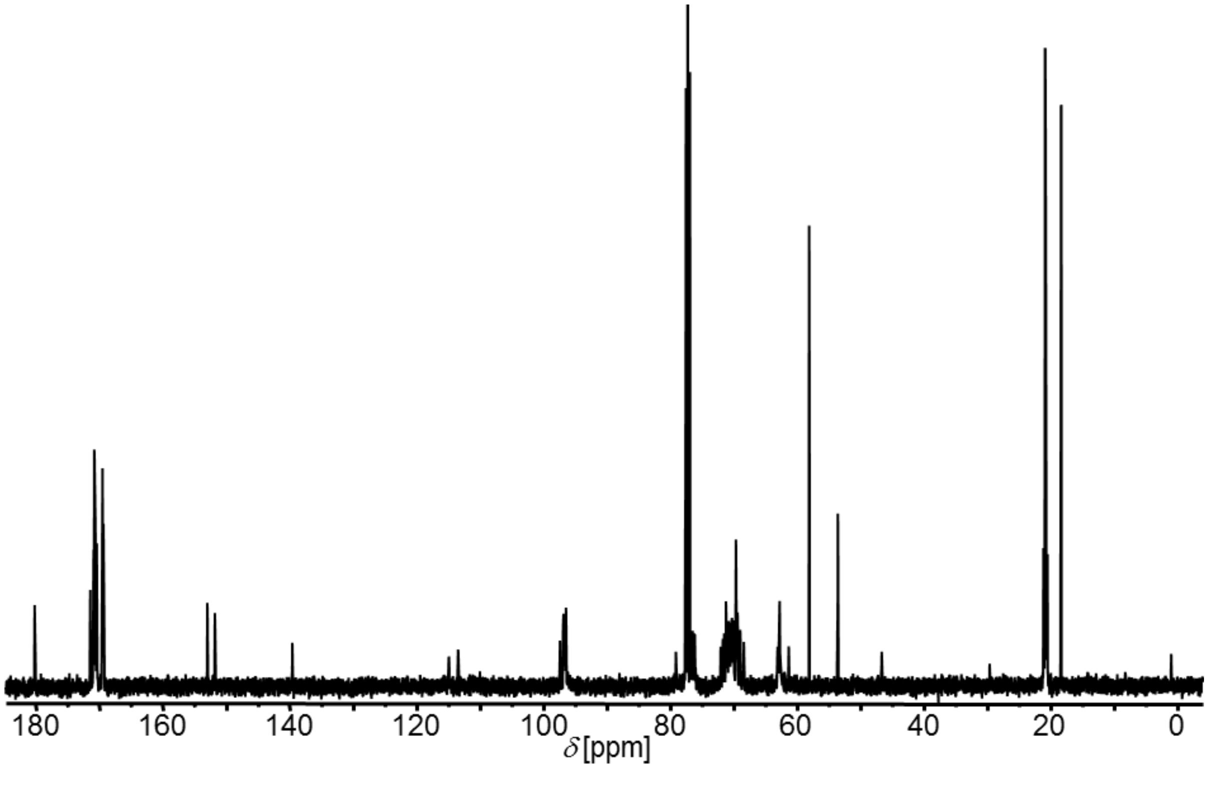

Figure S4: ^{13}C NMR spectrum (100.6 MHz, in $CDCl_3$ at 293 K) of **2**.

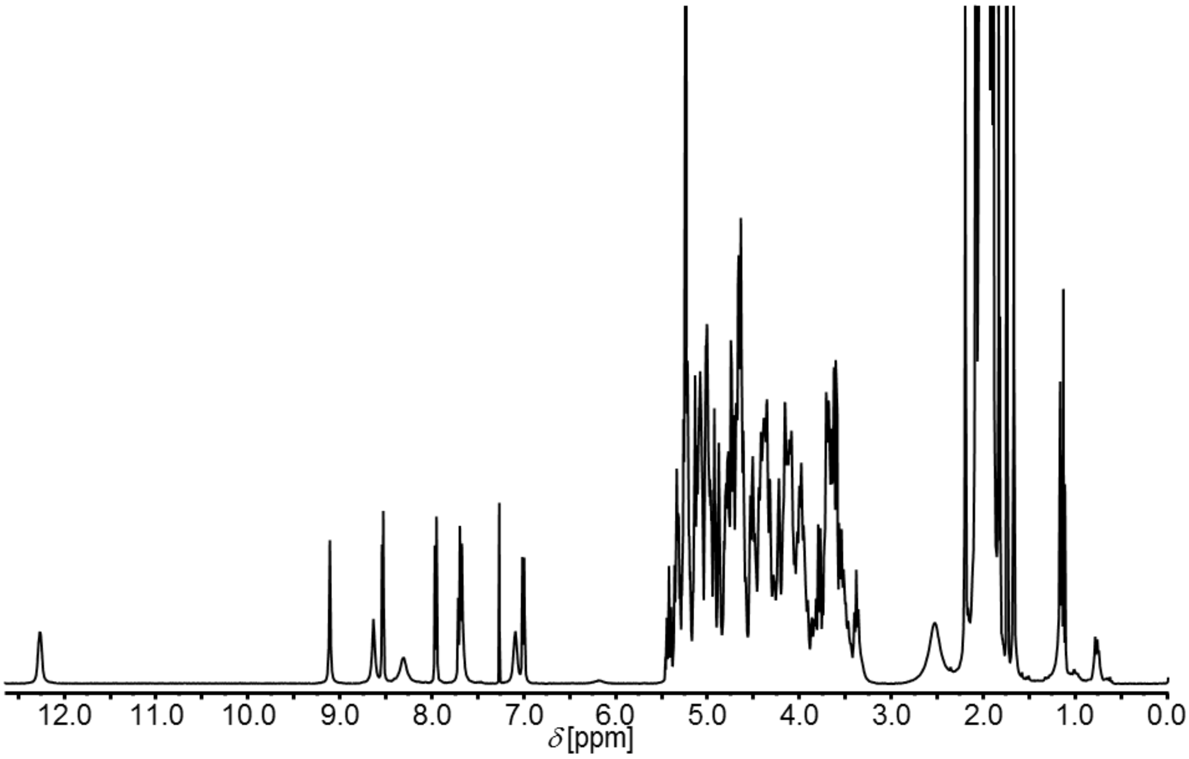

Figure S5: ^{1}H NMR spectrum (400.1 MHz, in $CDCl_3$ at 293 K) of **3**.

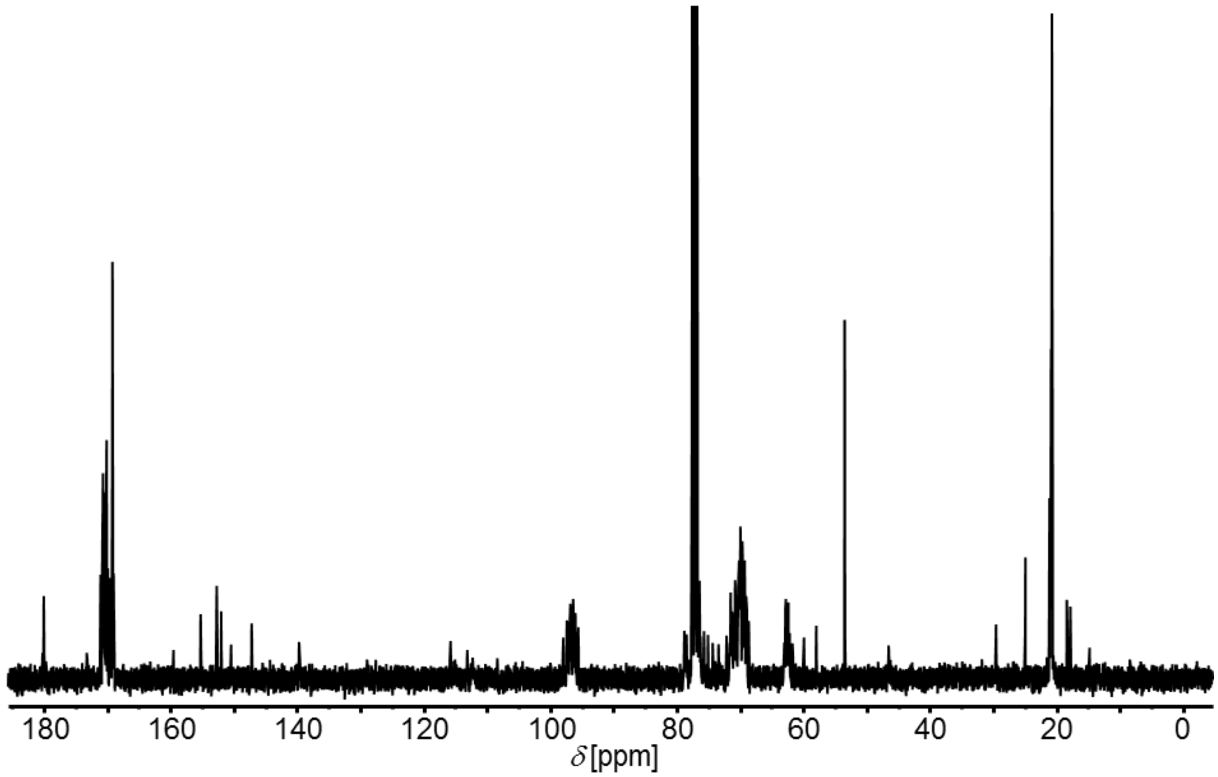

Figure S6: ^{13}C NMR spectrum (100.6 MHz, in $CDCl_3$ at 293 K) of **3**.

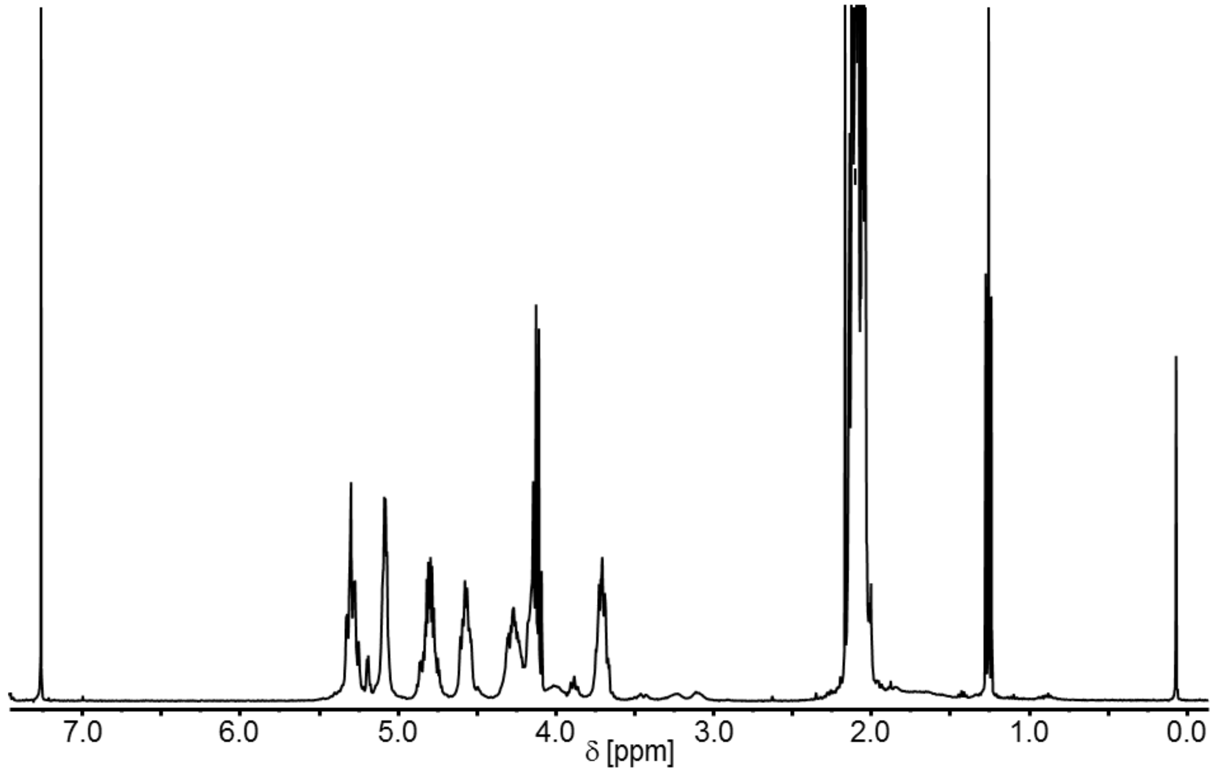

Figure S7: ^{1}H NMR spectrum (400.1 MHz, in $CDCl_3$ at 293 K) of **21**.

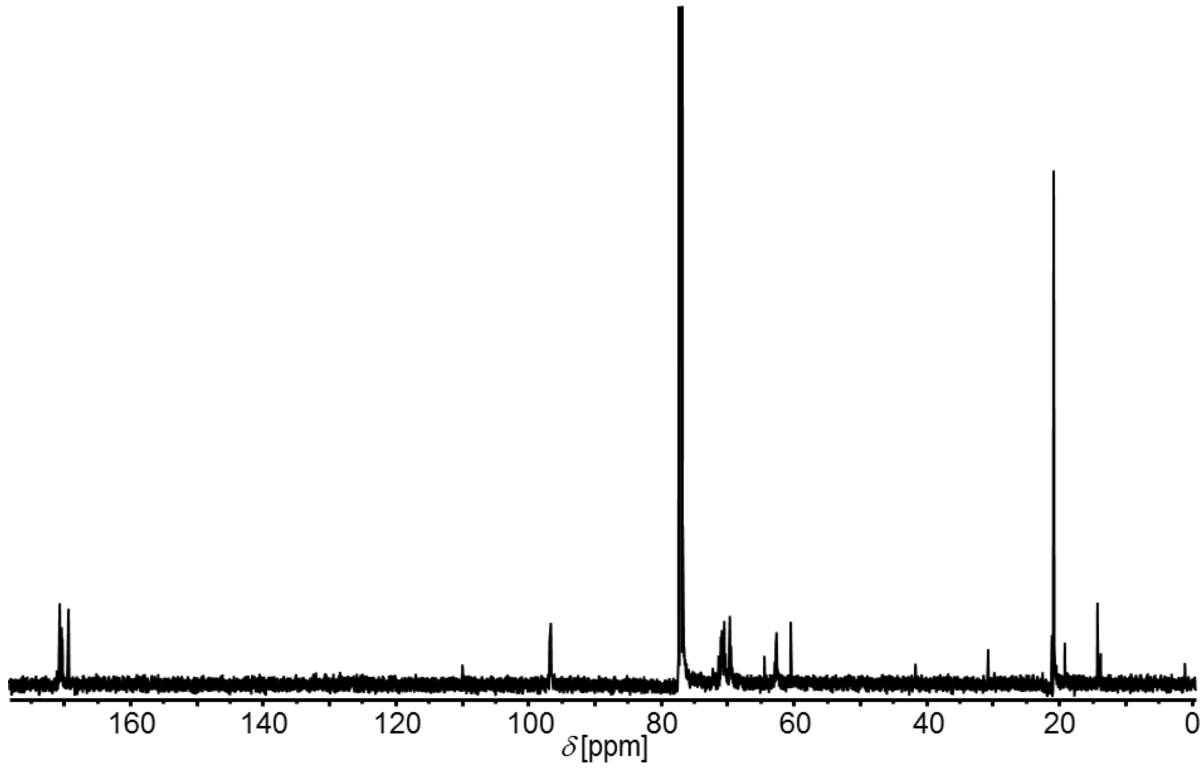

Figure S8: ^{13}C NMR spectrum (100.6 MHz, in $CDCl_3$ at 293 K) of **21**.

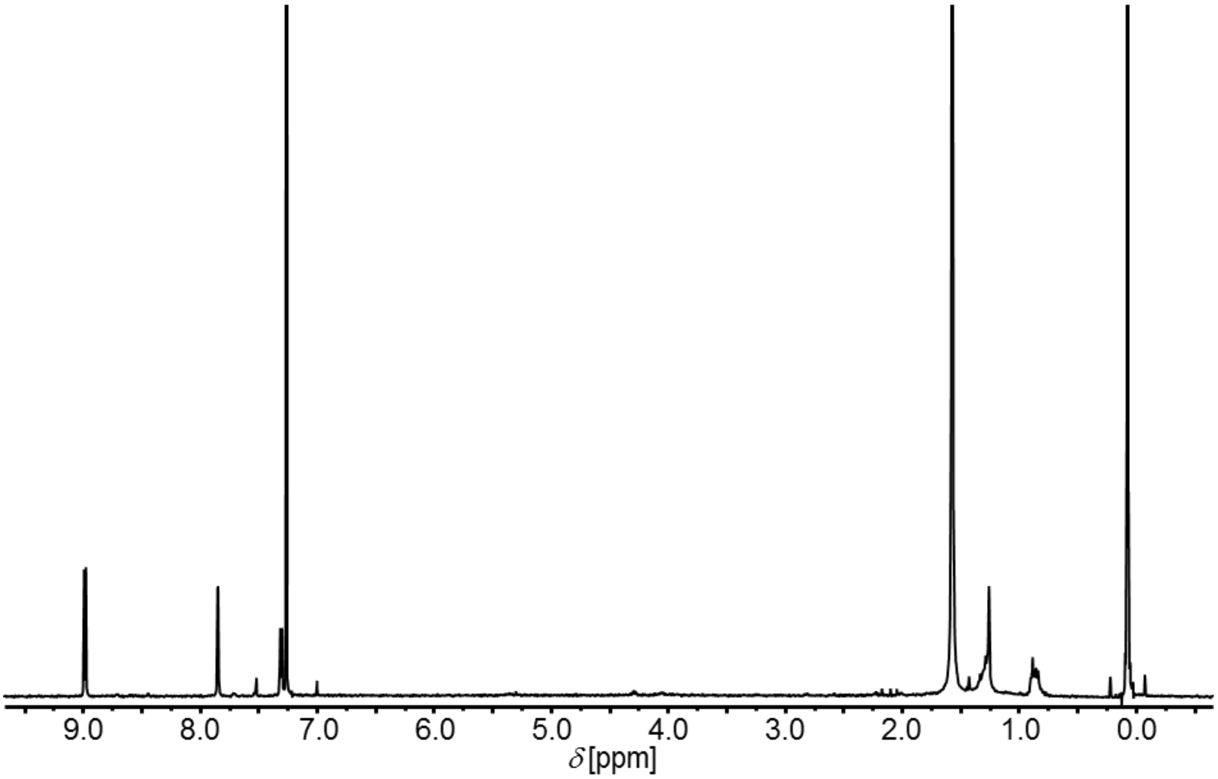

Figure S9: ^{1}H NMR spectrum (400.1 MHz, in $CDCl_3$ at 293 K) of [$(CO)_3$Re(**14**)Cl].

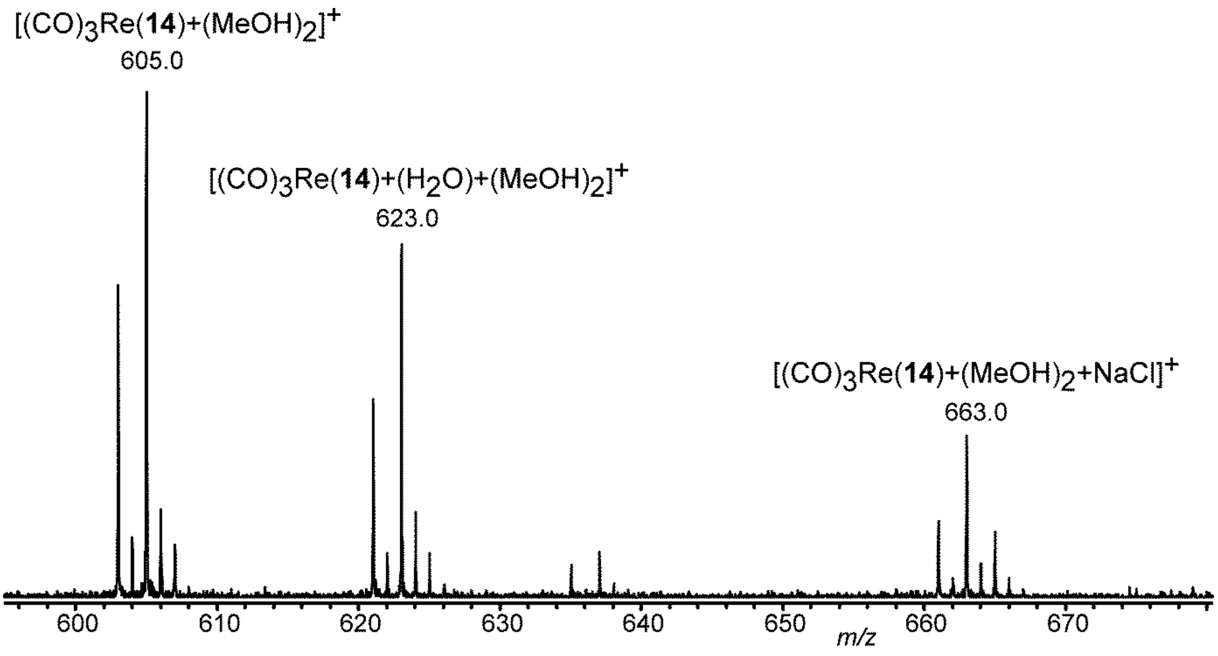

Figure S10: ESI–MS (positive mode, sprayed from benzene/acetonitrile 1:1) of [$(CO)_3$Re(**14**)Cl].

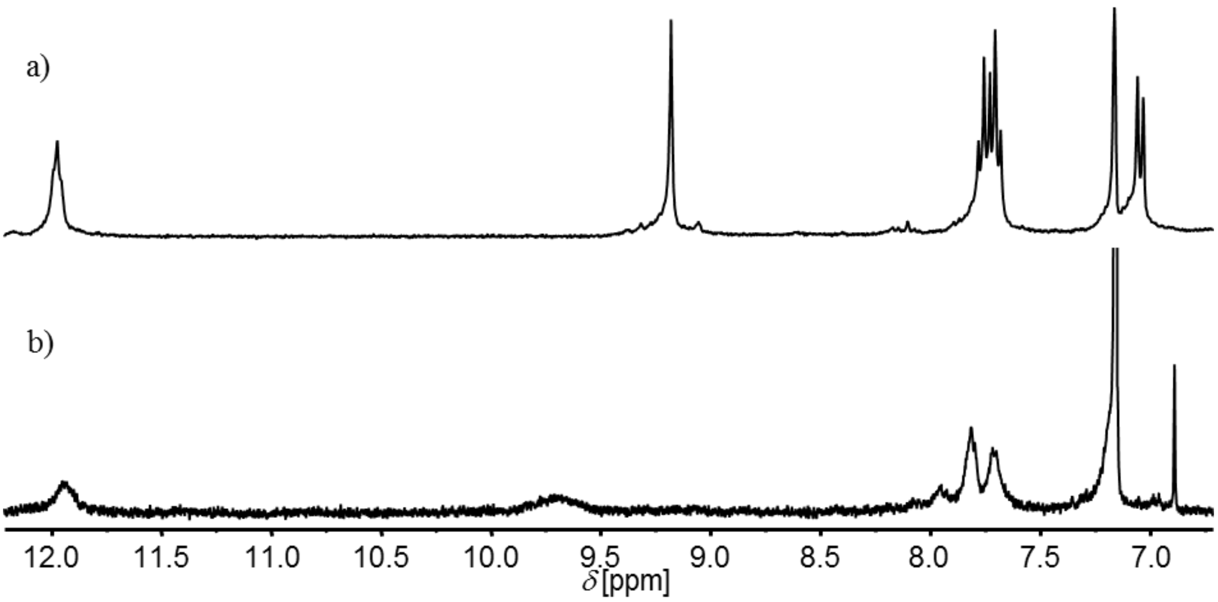

Figure S11: Aromatic region of the ^{1}H NMR spectra (100.6 MHz, 400.1 MHz, 293 K, benzene-d_6/acetonitrile-d_3 1:1) of a) **2** and b) [Cu(**2**)]PF_6.

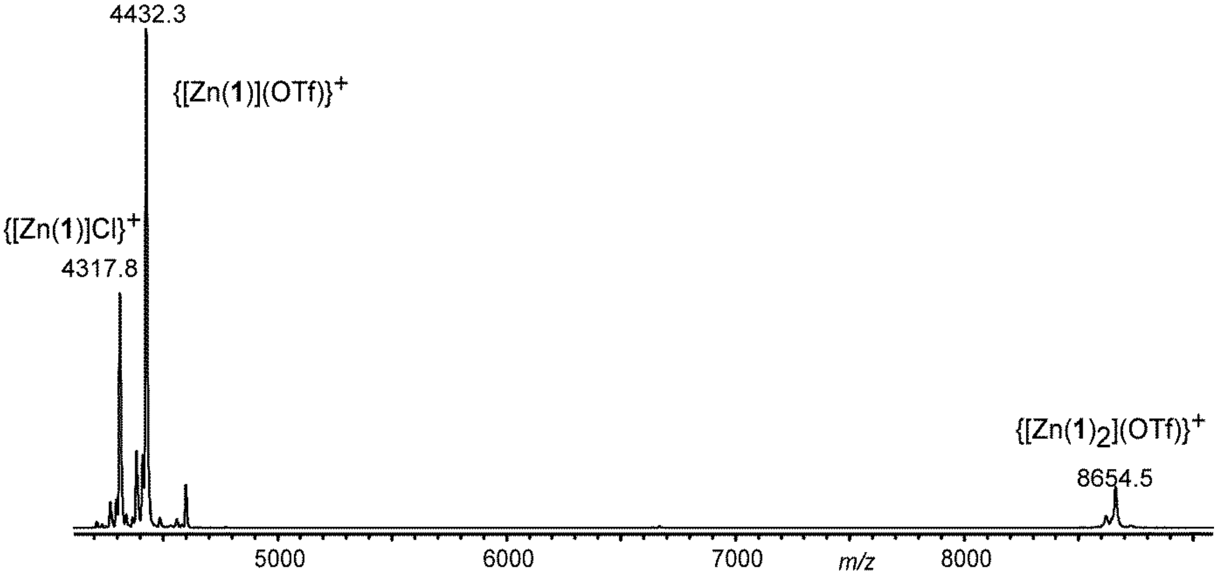

Figure S12: MALDI–MS (sample prepared from benzene/acetonitrile (1:1) solution using DCTB as matrix) of [Zn(**1**)$_2$](OTf)$_2$.

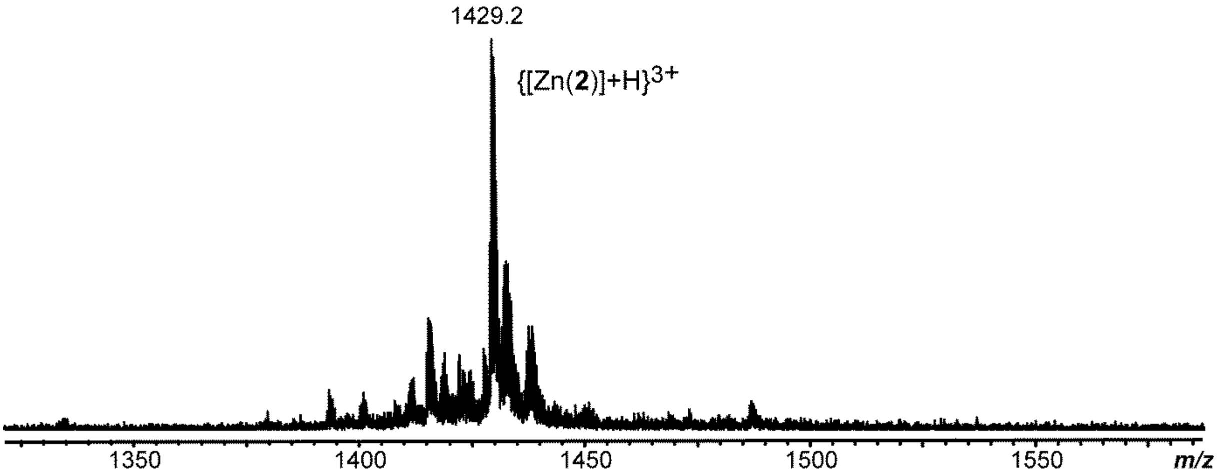

Figure S13: ESI–MS (positive mode, sprayed from benzene/acetonitrile 1:1) of $[Zn(\mathbf{2})](OTf)_2$.

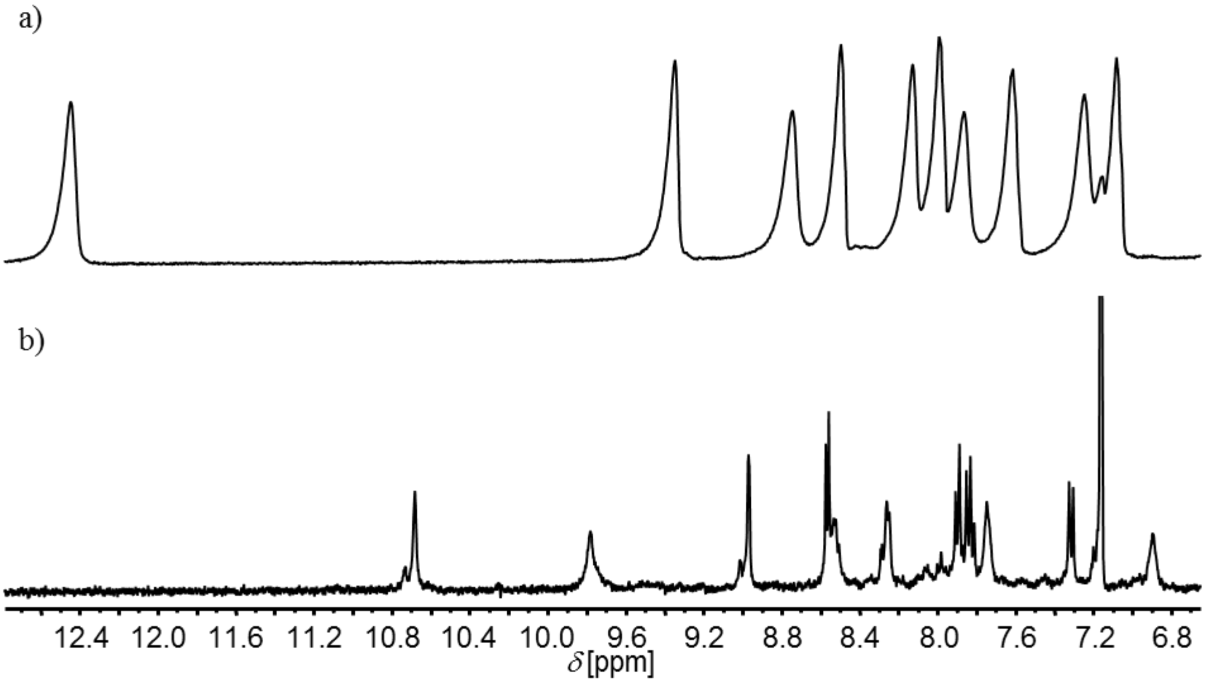

Figure S14: Aromatic region of the ^{1}H NMR spectra (100.6 MHz, 400.1 MHz, 293 K, benzene-d_6/acetonitrile-d_3 1:1) of a) **3** and b) a 1:2 mixture of $Zn(OTf)_2$ and **3**.

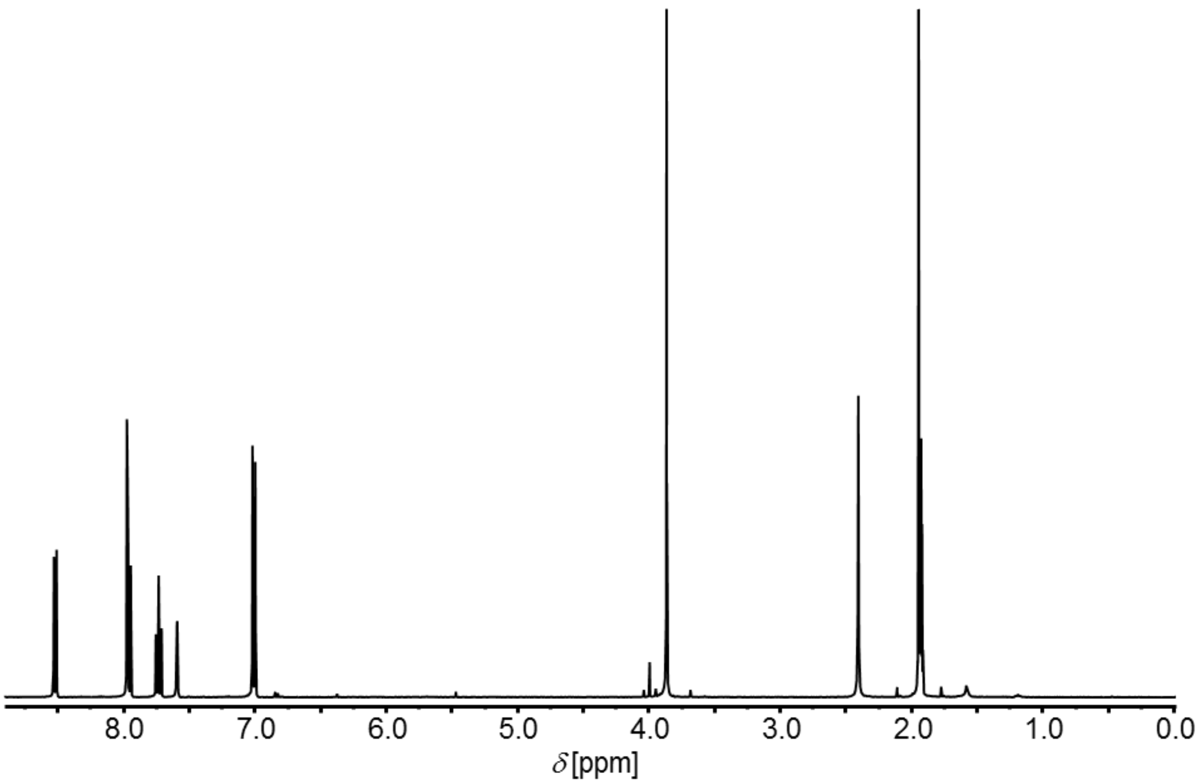

Figure S15: ^{1}H NMR spectrum (100.6 MHz, 400.1 MHz, 293 K, benzene-d_6/acetonitrile-d_3 1:1) of a 1:1 mixture of $CuPF_6$ and **22**.

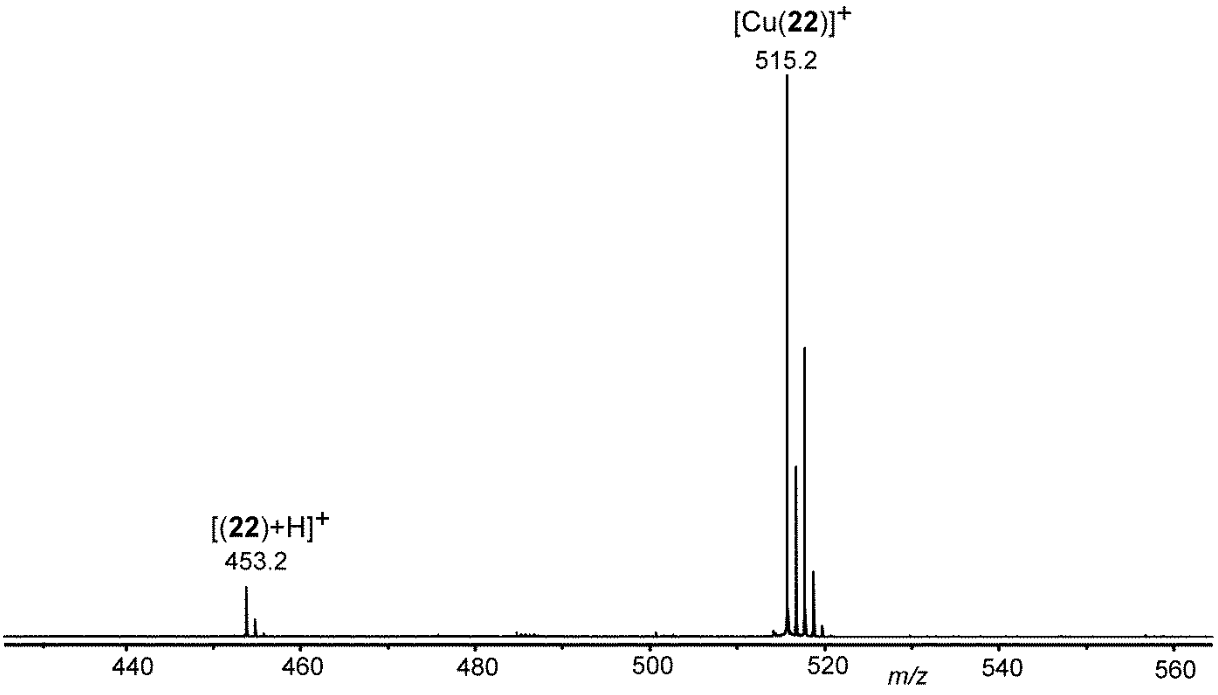

Figure S16: MALDI–MS (sample prepared from a benzene/acetonitrile (1:1) solution using DCBT as matrix) of [Cu(**22**)]PF_6.

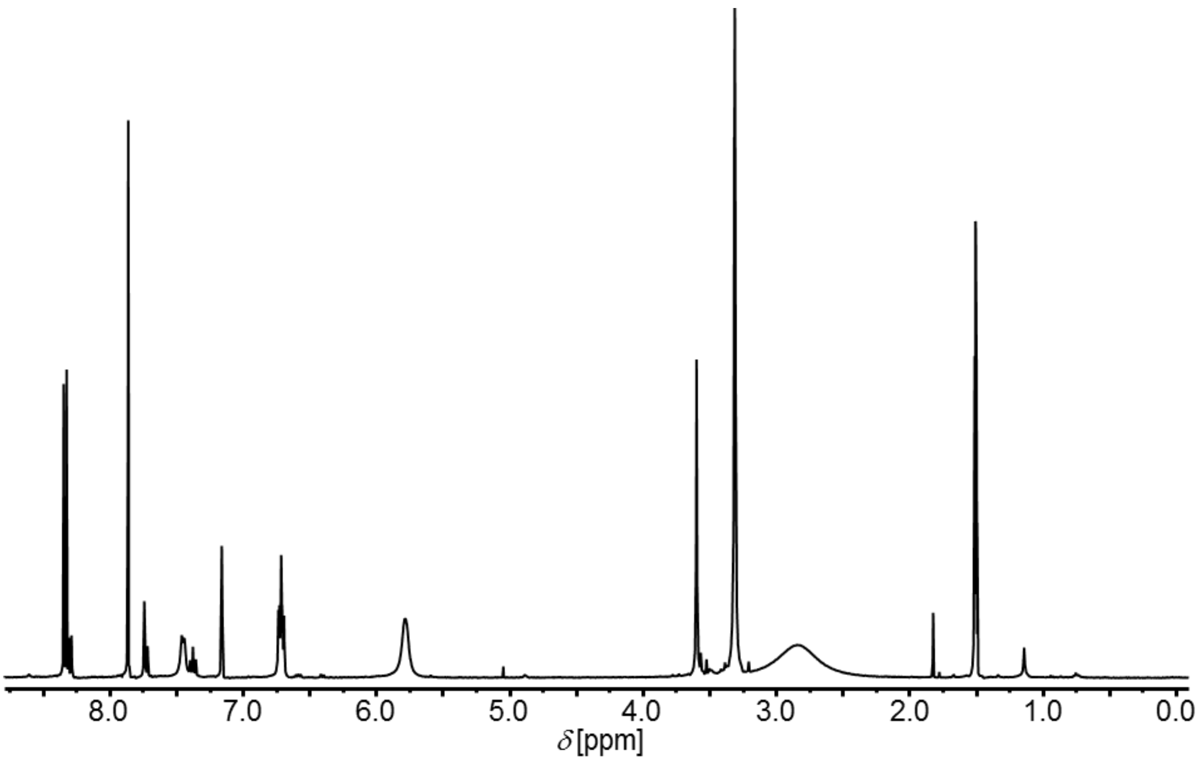

Figure S17: ^{1}H NMR spectrum (100.6 MHz, 400.1 MHz, 293 K, benzene-d_6/acetonitrile-d_3 1:1) of a 1:1 mixture of $Zn(OTf)_2$ and **22**.

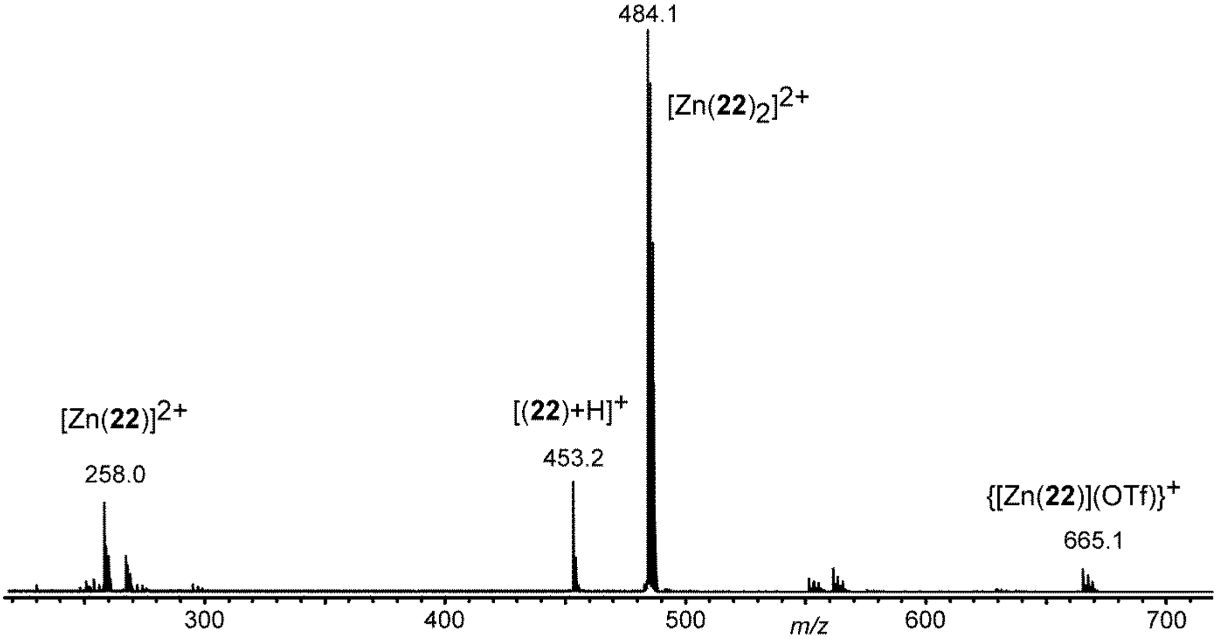

Figure S18: ESI–MS (positive mode, sprayed from benzene/acetonitrile 1:1) of $[Zn(\mathbf{22})](OTf)_2$.

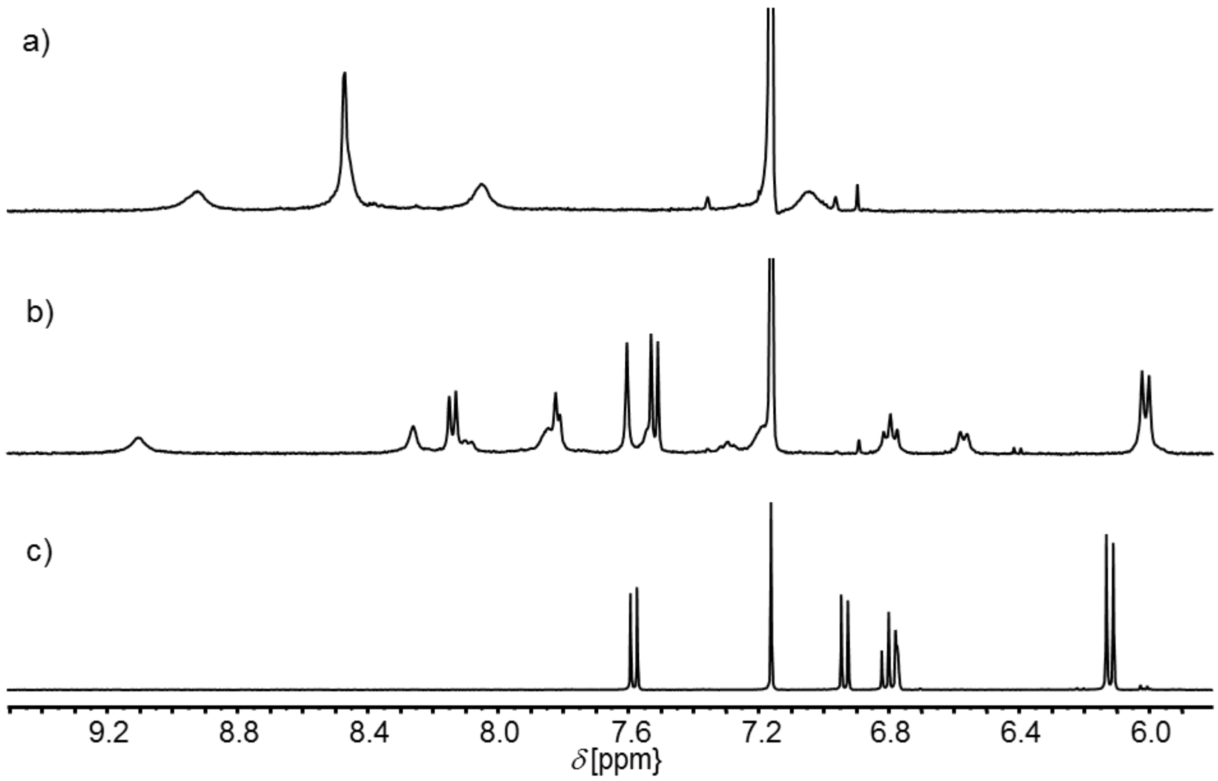

Figure S19: Aromatic region of the ^{1}H NMR spectra (100.6 MHz, 400.1 MHz, 293 K, benzene-d_6/acetonitrile-d_3 1:1) of a) **1**, b) a 1:1:1 mixture of $CuPF_6$, **1**, and **22**, and c) **22**.

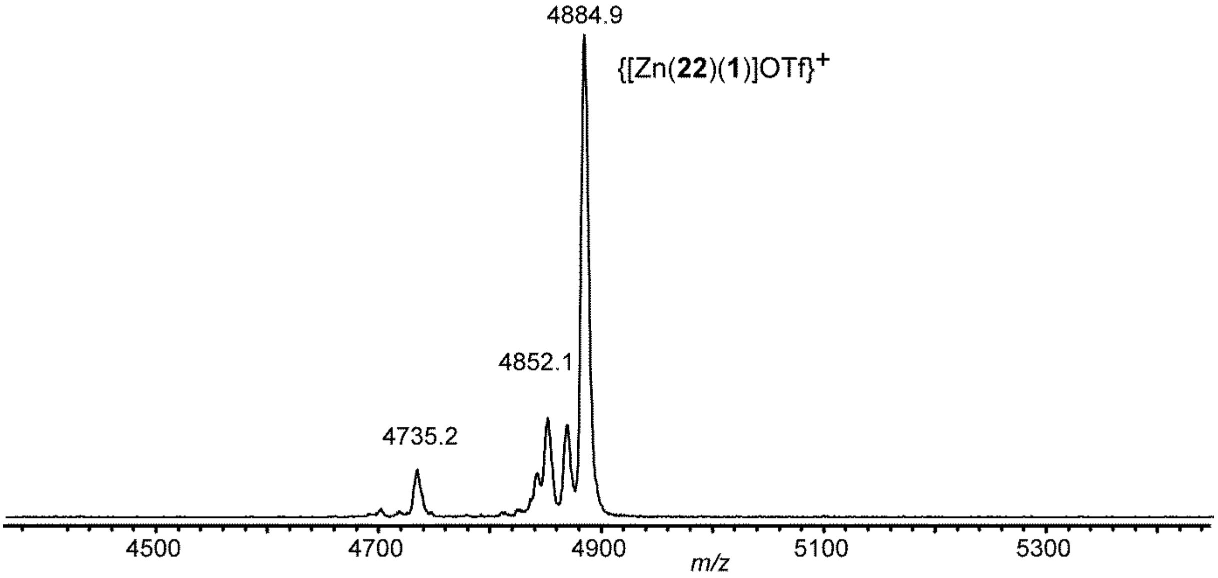

Figure S20: MALDI–MS (sample prepared from a benzene/acetonitrile (1:1) solution using DCTB as matrix) of [Zn(**22**)(**1**)]$(OTf)_2$.
